# Increased peritoneal TGF-β1 is associated with ascites-induced NK-cell dysfunction and reduced survival in high-grade epithelial ovarian cancer

**DOI:** 10.3389/fimmu.2024.1448041

**Published:** 2024-09-23

**Authors:** Ralph J. A. Maas, Janneke S. Hoogstad-van Evert, Iris M. Hagemans, Jolanda Brummelman, Diede van Ens, Paul K. J. D. de Jonge, Laura Hooijmaijers, Shweta Mahajan, Anniek B. van der Waart, Charlotte K. J. C. Hermans, Janne de Klein, Rob Woestenenk, Antonius E. van Herwaarden, Nicolaas P. M. Schaap, Somayeh Rezaeifard, Daniele V. F. Tauriello, Petra L. M. Zusterzeel, Nelleke Ottevanger, Joop H. Jansen, Willemijn Hobo, Harry Dolstra

**Affiliations:** ^1^ Laboratory of Hematology – Department of Laboratory Medicine, Radboud University Medical Center, Nijmegen, Netherlands; ^2^ Department of Obstetrics and Gynecology Amphia, Breda, Netherlands; ^3^ Diagnostic Laboratory – Department of Laboratory Medicine, Radboud University Medical Center, Nijmegen, Netherlands; ^4^ Department of Hematology, Radboud University Medical Center, Nijmegen, Netherlands; ^5^ Department of Cell Biology, Radboud University Medical Center, Nijmegen, Netherlands; ^6^ Department of Obstetrics and Gynecology, Radboud University Medical Center, Nijmegen, Netherlands; ^7^ Department of Medical Oncology, Radboud University Medical Center, Nijmegen, Netherlands

**Keywords:** ovarian cancer, ascites, natural killer (NK) cells, TGF-β, tumor microenvironment

## Abstract

Natural killer (NK) cell therapy represents an attractive immunotherapy approach against recurrent epithelial ovarian cancer (EOC), as EOC is sensitive to NK cell-mediated cytotoxicity. However, NK cell antitumor activity is dampened by suppressive factors in EOC patient ascites. Here, we integrated functional assays, soluble factor analysis, high-dimensional flow cytometry cellular component data and clinical parameters of advanced EOC patients to study the mechanisms of ascites-induced inhibition of NK cells. Using a suppression assay, we found that ascites from EOC patients strongly inhibits peripheral blood-derived NK cells and CD34+ progenitor-derived NK cells, albeit the latter were more resistant. Interestingly, we found that higher ascites-induced NK cell inhibition correlated with reduced progression-free and overall survival in EOC patients. Furthermore, we identified transforming growth factor (TGF)-β1 to correlate with ascites-induced NK cell dysfunction and reduced patient survival. In functional assays, we showed that proliferation and anti-tumor reactivity of CD34+ progenitor-derived NK cells are significantly affected by TGF-β1 exposure. Moreover, inhibition of TGF-β1 signaling with galunisertib partly restored NK cell functionality in some donors. For the cellular components, we showed that the secretome is associated with a different composition of CD45+ cells between ascites of EOC and benign reference samples with higher proportions of macrophages in the EOC patient samples. Furthermore, we revealed that higher TGF-β1 levels are associated with the presence of M2-like macrophages, B cell populations and T-regulatory cells in EOC patient ascites. These findings reveal that targeting TGF-β1 signaling could increase NK cell immune responses in high-grade EOC patients.

## 1 Introduction

The prognosis of epithelial ovarian carcinoma (EOC) is poor, with a five-year overall survival (OS) of 32% in advanced-stage disease (1). Since EOC patients frequently have vague symptoms, 70% of patients presents with advanced stage III or IV disease. Often EOC is accompanied by ascites, an abnormal accumulation of fluid in the peritoneal cavity due to lymphatic obstruction and increased vascular permeability (2). Ascites promotes tumor cell growth and invasion, and contains immune cells such as lymphocytes and macrophages as well as stromal/tissue-resident cells (3, 4). Furthermore, ascites comprises a large variety of soluble factors released by these cells, thereby playing an important role in influencing anti-tumor immunity within the abdominal cavity (5). The amount of ascites, as well as the cellular and soluble factor make-up varies from patient to patient (6). A more detailed insight into this immune environment could aid in understanding which dominant factors contribute to suppression of anti-tumor immune responses in EOC patients.

Immunotherapy could be a complementary approach to existing EOC treatments, as it is considered to be an immunogenic tumor type. The presence of tumor-infiltrating lymphocytes (TILs) positively correlates with survival, whereas the presence of regulatory T cells (Tregs) is associated with decreased survival (7–9). As ovarian tumor cells often downregulate MHC class I while expressing natural killer (NK) cell activating ligands, they are prone to NK cell-mediated immune responses (10–12). Furthermore, a high NK cell percentage within ascites correlates with better survival of EOC patients (13). Hence, boosting NK cell immunity through immunotherapeutic strategies may improve outcome in advanced EOC patients. Nevertheless, NK cell function can be dampened by immunosuppressive cells and soluble mediators within the ascites. For instance, the NK cell inhibitory effect of TGF-β1 and IL-10 in ascites was already described in the early 90’s (14, 15). Furthermore, several studies have shown that exposure to soluble or cell bound B7-H6, PVR (CD155) and MIC-A/B results in downregulation of the NK cell activating receptors NKp30, DNAM-1 and NKG2D (16–20).

To further elucidate mechanisms impairing NK cell function in the local EOC environment, we combined NK-cell functional data, soluble factor analysis, high-dimensional flow cytometry assessment of cellular components and clinical parameters of 31 advanced EOC patients and 16 benign peritoneal fluids in integrated analyses. We found strong inhibitory effects on healthy donor-derived NK cells by EOC patient ascites, that were correlated to patient progression-free (PFS) survival, overall survival (OS) and serum CA-125 levels. Using soluble factor analysis, we revealed that TGF-β1 was a discrete inhibitory factor correlating to ascites-induced NK cell dysfunction. Furthermore, we showed that blocking TGF-β1 signaling using galunisertib partially rescued NK cell functionality. Galunisertib is a small molecule inhibitor that binds the intracellular serine-threonine kinase domain of TGFβR1 and thereby prevents phosphorylation SMAD2/3 signaling and nuclear translocation which disrupts TGFβ1-mediated signaling Moreover, using multicolor flow cytometry we identified tumor-associated macrophages (TAMs), B cell populations and Tregs to be the main cell types associated with the suppressive factor profile found in ascites of advanced EOC patients. Altogether, our data provide a rationale for anti-TGF-β1 treatment strategies to augment NK cell responses in high-grade EOC patients.

## 2 Materials and methods

### 2.1 Patient samples

EOC patient ascites fluid samples of patients with FIGO stage III or IV high-grade serous EOC were collected after written informed consent at first surgery at the Radboud University Medical Center or Canisius Wilhelmina Hospital. All high grade FIGO stage III or IV EOC patients with ascites were asked to participate in this study. Ascites was collected at diagnosis or during primary surgery. Diagnosis of high grade EOC was based on histology. Study approval was given by the Regional Committee for Medical Research Ethics (CMO 2018-4845) and performed according to the Code for Proper Secondary Use of Human Tissue (Dutch Federation of Biomedical Scientific Societies, www.federa.org). The PFS and OS at time of analysis, CA-125 levels (serum and peritoneal) and treatment status are shown for the patient cohort in **Supplementary Table 1** and individual patients in **Supplementary Table 2**. The median time of follow up was 21.6 months (16.5-53.8 months). Samples of abdominal washing fluid or free fluid from patients without any malignancy, planned for surgery of a benign cyst were used as a relevant reference sample. We chose both abdominal washings and ascites of benign indications to be able to visualize the differences in soluble factors in a cancer microenvironment versus a benign one. Benign samples were excluded in case of signs of active infection and had a serous cystadenoma, endometrioma, mucinous cystadenoma or fibroma (**Supplementary Table 3**). Ascites samples were filtered using a 100 µm filter and centrifuged. Cell-free ascites supernatant was stored at -20°C for secretome analysis and NK cell activity assays. Cells were resuspended in phosphate buffered saline (PBS) for subsequent mononuclear cell isolation using a Ficoll-Hypaque (1.077 g/mL; GE Healthcare, 17–1440–03) density gradient. Cells were cryopreserved in Iscove’s Modified Dulbecco’s medium (IMDM; Gibco, #12440061) supplemented with 10% dimethyl sulfoxide (DMSO) and 10% fetal calf serum (FCS, Integro) and used after thawing.

### 2.2 NK cell isolations

Peripheral blood mononuclear cells (PBMCs) were obtained from healthy donor buffy coats (Sanquin Blood Supply Foundation, Nijmegen, the Netherlands) by density gradient Ficoll-Hypaque centrifugation and frozen for phenotypic analysis (n=14, Mean age (SD) 47(± 17) years) or used fresh for subsequent NK cell isolation used in functional assays. Peripheral blood (PB)-NK cells were isolated from PBMCs of healthy donors using a magnetic bead-based NK cell enrichment kit (StemCell Technologies, #19055) according to manufacturer’s instructions. All isolations resulted in ≥90% purity, as measured by flow cytometry.

### 2.3 HPC-NK cell culture

CD34+ hematopoietic progenitor cells (HPCs) were isolated from umbilical cord blood (UCB). UCB was collected at caesarean sections after informed consent (approved by the Radboudumc Committee for Medical Research Ethics CMO 2014/226), in accordance with institutional guidelines and regulations, and the Declaration of Helsinki. CD34+ HPCs were isolated from mononuclear cells after Ficoll–Hypaque density-gradient centrifugation and subsequent CD34-positive immunomagnetic bead selection (Miltenyi Biotec, 130046702). CD34+ HPCs were expanded and differentiated into HPC-NK cells in a 5-week culture protocol, as described previously (21). HPC-NK cells (≥70% CD56+) were used immediately after culture, or after cryopreservation followed by 5-8 days of culture in NK MACS basal medium plus supplement (NK MACS, Miltenyi Biotec, 130–114-429) containing 10% human serum (HS), 50 ng/ml recombinant human (rh)IL-15 (Immunotools, 11340155) and 0.2 ng/ml rhIL-12 (Miltenyi Biotec, 130–096-704).

### 2.4 Tumor cell culture

SKOV-3 (RRID: CVCL_0532) was cultured in Roswell Park Memorial Institute medium 1640 (RPMI; Gibco, #11875091) supplemented with 10% (FCS; Integro). K562 (RRID: CVCL_0004) was cultured in IMDM (Gibco, #12440061) containing 10% FCS. Cell lines were tested for mycoplasma contamination with MycoAlertTM Mycoplasma Detection Kit (Lonza, #LT07-418) every six months. Cell lines were purchased from the ATCC and cultured for a maximum of 3 months.

### 2.5 Luminex, ELISA, Flow cytometry and NK cell functionality assays

Ascites of EOC and benign patients was assessed for soluble factors by Luminex and ELISA. Flow cytometry and high-dimensional data analysis was performed as described in the supplementary information. NK functionality assays were performed with peripheral blood-derived and CD34+ progenitor-derived NK cells. For details see Supplementary information.

### 2.6 Statistics

Data analysis was conducted using Prism software (GraphPad, version 5.03 for Windows). For normally distributed data, the Student t-test (paired or unpaired) or One-way ANOVA (with or without repeated measure) was used, as stated in the figure legends. Non-normally distributed data was tested with a Wilcoxon signed-rank test, Mann-Whitney test, Kruskal-Wallis or Friedman test, as indicated in the figure legends. To identify correlations between variables (e.g. frequency of immune cell subpopulations and soluble factors), Spearman correlation analysis was performed and visualized in R using the corrplot package (version 0.84). A p-value of <0.05 was considered statistically significant.

## 3 Results

### 3.1 Inhibition of NK cell functionality by EOC ascites is correlated with reduced patient survival

Soluble factors in ascites have been demonstrated to impair immune cell function within the tumor microenvironment (TME) of EOC patients. To assess the inhibitory effect on NK cell function, we pre-treated PB or hematopoietic progenitor-derived (HPC) NK cells with various amounts of patient-derived ascites in the presence or absence of IL-15. After overnight exposure, NK cells were challenged with tumor cells and evaluated for CD107a-based degranulation and IFN-γ response. Dose-dependent inhibitory effects were observed when NK cells were pre-treated with increasing amounts of ascites both with or without rhIL-15 (**Supplementary Figure 1**), whereas the presence of ascites had no negative effects when co-incubated during the 4h tumor cell stimulation without pre-incubation (**Supplementary Figure 2**). As the most prominent effects were seen with the presence of 50% ascites during the 16h pre-treatment, we used this concentration for all subsequent PB-NK and HPC-NK inhibition assays.

**Figure 1:**
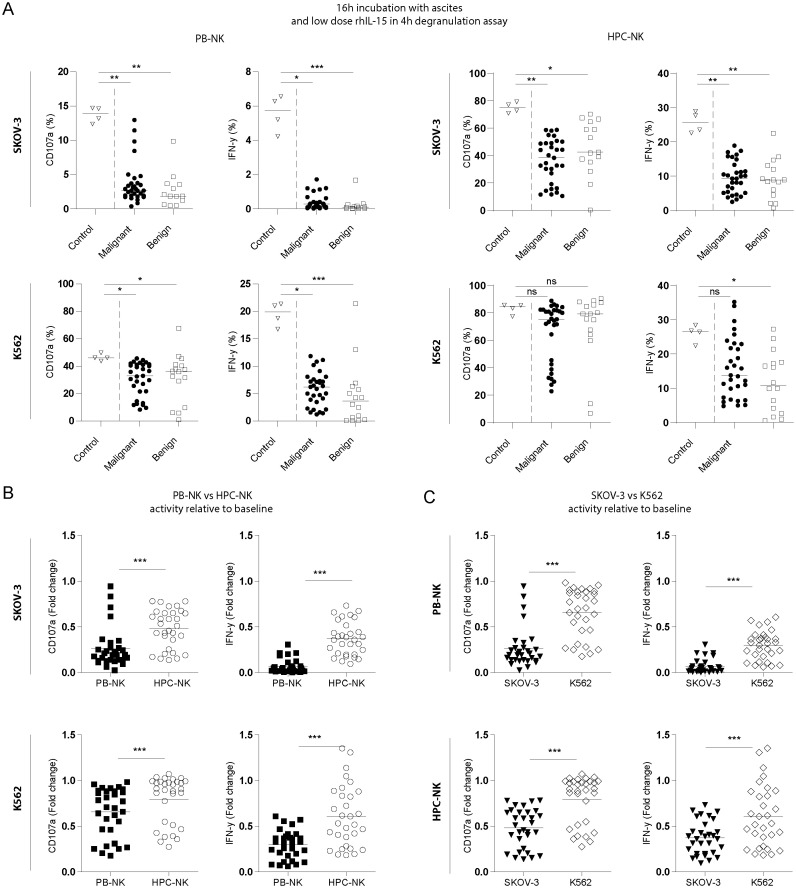
Ascites from EOC patients inhibits NK cell activity. **(A)** Percent positive CD107a and IFN-γ PB-derived (left) and HPC-derived (right) NK cells stimulated with SKOV-3 (top) or K562 (bottom) target cells in the presence of PBS (open triangles), EOC patient ascites (filled circles, n=31) or benign fluid (open squares, n=15). Cells were incubated overnight with aforementioned fluids with addition of 1nM rhIL-15 and challenged with target cells for 4 hours. For control quadruplicates are shown, and for EOC patient and benign control fluids the average of duplicates are depicted and were used for statistics. Kruskal-Wallis with Dunn’s Multiple Comparison Test was used for statistical analysis, * p < 0.05, ** p < 0.01 and *** p < 0.001. **(B)** and **(C)** Fold change CD107a and IFN-γ inhibition of the same assay compared to control of EOC patient ascites (n=31) with for **(B)** HPC-derived (open circles) and PB-derived (filled squares) NK cells stimulated with SKOV-3 (top) and K562 (bottom) or for **(C)** stimulated with SKOV-3 (filled triangles) and K562 (open diamonds) on PB-NK (top) or HPC-NK cells (bottom). Wilcoxon signed-rank test was used for statistical analysis, *** p < 0.001 **(C)**.

After defining the optimal conditions to demonstrate the inhibitory effects in the *in vitro* assay, we tested the inhibitory properties of peritoneal fluids from 31 EOC patients and 16 benign reference patients for inhibition of NK cells towards SKOV-3 or K562 target cells with both NK cell sources (**Figure 1A**). We found that peritoneal fluids, regardless of their origin, strongly inhibited NK cell functionality (degranulation activity and IFN-γ production) in all tested conditions compared to the PBS control condition. Only in the K562-stimulated HPC-NK cells the effect on CD107a degranulation was not statistically significant (**Figure 1A**). Viability of NK cells in this assay was 82 ± 7% for HPC-NK cells and 97 ± 3% for PB-NK cells across all different conditions indicating that the large volume of non-media does not affect NK cell viability. Interestingly, the different NK-cell sources were not equally affected, as HPC-NK cells were significantly more resistant to ascites-mediated suppression than PB-NK cells for all tested parameters (**Figure 1B**). Furthermore, ascites-mediated suppression had more impact on NK-cell responses towards SKOV-3 target cells than against MHC class I-negative K562 cells (**Figure 1C**).

**Figure 2:**
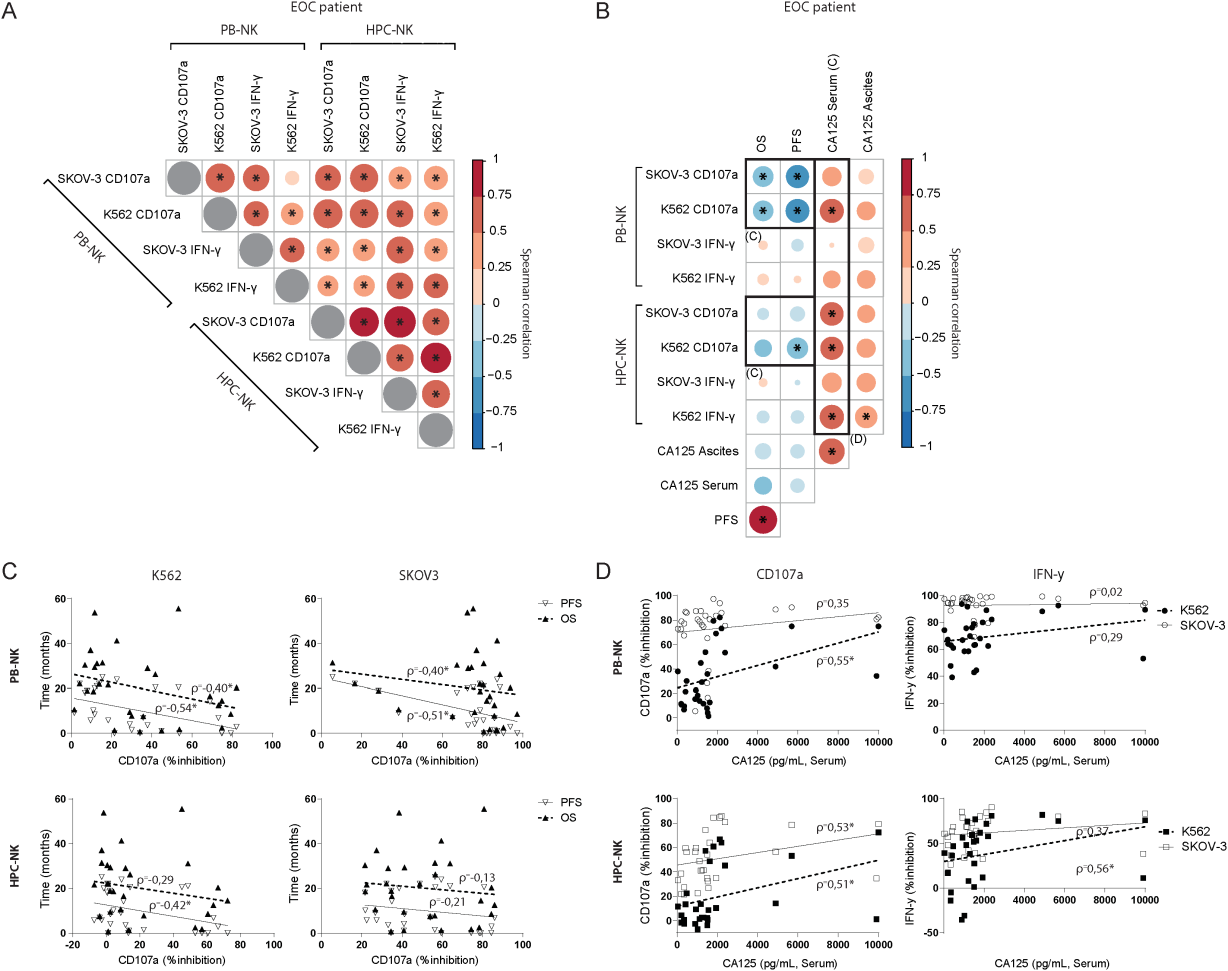
Inhibitory properties of ascites on NK cells correlates to patient survival and CA-125 levels. **(A, B)** Spearman correlograms of mean fold change of suppression by EOC patient ascites between all tested conditions, i.e. HPC-NK vs PB-NK, SKOV-3 vs K562 and CD107a vs IFN-γ **(A)**, or those conditions versus OS, PFS, CA-125 (serum and ascites) **(B)**; with color intensity and circle size indicating the Spearman’s rank correlation coefficient between biomarkers, and * denoting p < 0.05. **(C)** Scatter plots illustrating the relationship between PFS and OS in months versus PB-NK CD107a (top) or IFN-γ (right) response to SKOV-3 (left) and K562 (right). Spearman’s rank correlation coefficient (ρ) shown, * p < 0.05 **(D)** Scatter plots illustrating the relationship between CD107a (left) or IFN-γ (right) and CA-125 serum levels in PB-NK (top) and HPC-NK cells (bottom).

The inhibitory effects were consistent across different parameters between the different functional read-outs (i.e. PB-NK vs. HPC-NK, SKOV-3 vs. K562, and CD107a vs. IFN-γ, **Figure 2A**). Next, we assessed the correlation between ascites-mediated NK cell dysfunction and clinical parameters of the advanced EOC patients (**Figure 2B**). The mean age of the selected EOC patient cohort (n=31) was 63 ± 11 years and 53 ± 10 years for the benign reference group (n=12; excluding 4 patients of whom age and CA-125 levels were not available). The median OS and PFS of the advanced EOC patient cohort at time of analysis was 18.9 and 6.9 months, respectively, with seven of the patients still alive at time of analysis (**Supplementary Tables 1**, **2**). Interestingly, ascites-induced impairment of PB-NK cell reactivity against either K562 or SKOV-3 target cells was significantly correlated with lower PFS (**Figures 2B, C**; ρ= -0.54 for K562 and -0.51 for SKOV-3) and OS (ρ=-0.40 for both K562 and SKOV-3). Similarly, ascites-induced HPC-NK dysfunction in response to K562 targets was negatively associated with PFS (**Figures 2B, C**; ρ=-0.42). Furthermore, we observed a positive correlation between ascites-induced NK cell dysfunction and higher CA-125 levels in the serum of EOC patients (**Figures 2B, D**). CD107a degranulation activity was more strongly correlated to serum CA-125 levels for both PB-NK cells (ρ=0.35 and 0.55 for K562 and SKOV-3, respectively) and HPC-NK cells (ρ=0.53 and 0.51 for K562 and SKOV-3, respectively). For IFN-γ production only HPC-NK inhibition significantly correlated to both CA-125 serum and peritoneal levels (ρ=0.53 and 0.56, respectively; **Figure 2D**). Collectively, these data demonstrate that ascites from EOC patients contains soluble inhibitory factors that potently impair NK cell function, which significantly correlates to reduced survival and higher serum CA-125 levels.

### 3.2 High TGF-β1 concentration is correlated with EOC ascites-mediated NK cell dysfunction

**Figure 3:**
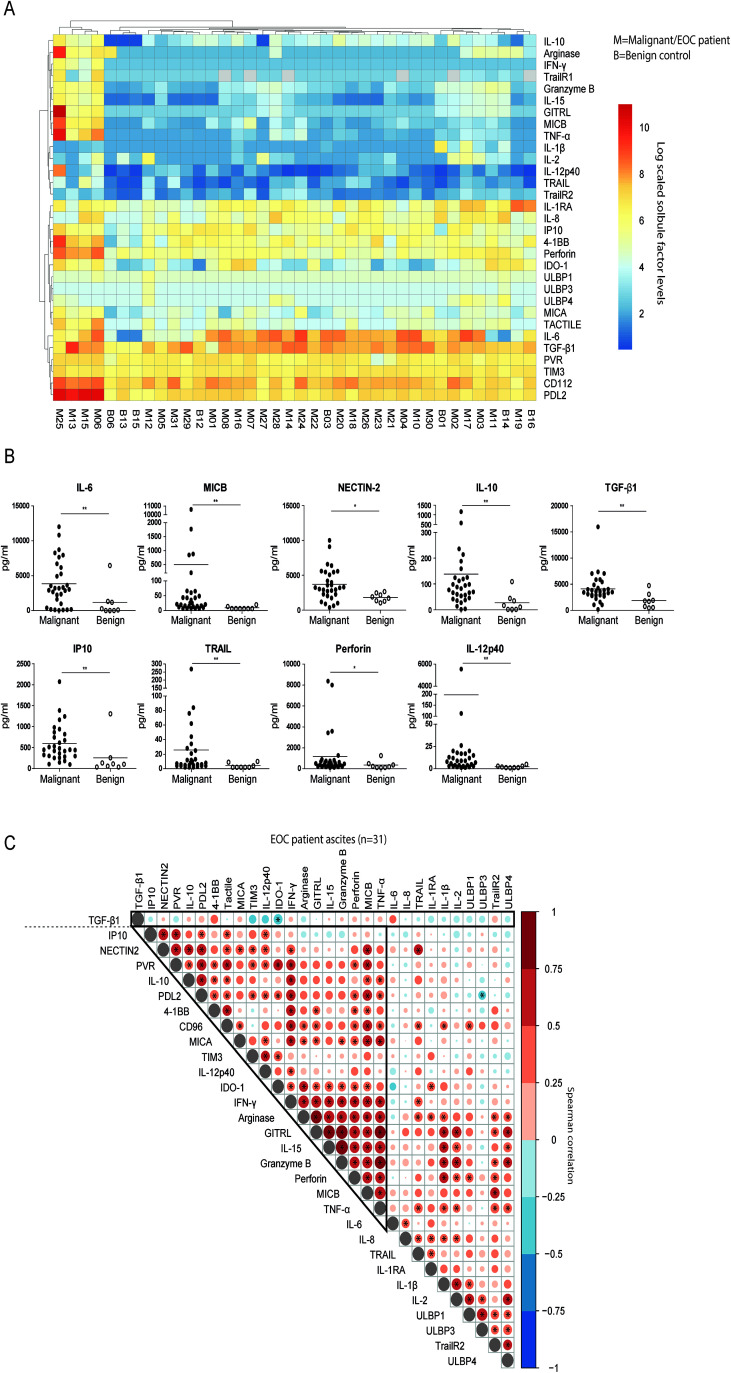
TGF-β1 as a discrete suppressive cytokine in EOC patient ascites. **(A)** Log scaled soluble cytokine levels by Luminex or ELISA on of EOC patient (n=31) and benign control (n=8) peritoneal fluids visualized in a heatmap. Each row represents a different cytokine, while columns represent patients or donors (M=malignant and B=benign). The log scaled cytokine level of patients or donors is reported and visualized with a color scale from blue (low levels) to red (high levels). **(B)** Cytokine levels of significantly different cytokines in the panel of EOC patient and benign control ascites fluids. A Mann-Whitney test was used for statistical analysis, * p < 0.05 and ** p < 0.01. **(C)** Spearman correlogram of mean fold change of suppression by EOC patient ascites in all tested condition and clinical parameters versus cytokine levels. A heat map is used to indicate the Spearman’s rank correlation coefficient (ρ) of associations between biomarkers. Red indicates a positive correlation, and blue indicates a negative correlation. * p < 0.05.

After establishing that soluble factors in ascites can potently induce NK cell dysfunction, we sought to find dominant inhibitory factors in ascites that could impair NK cell reactivity. For this, we examined peritoneal fluids in 25-plex Luminex and ELISAs in parallel with NK cell suppression assays (**Figure 3A**). Although we observed that the general cytokine profiles of benign and EOC patient ascites fluids were mostly similar, there were individual soluble factors that were significantly different (**Figures 3A, B**). We verified that IL-6 concentration, a cytokine previously associated with poor outcome in EOC patients (22), was significantly elevated in EOC patient ascites compared to benign ascites. Furthermore, soluble ligands for activating NK cell receptors such as MIC-B (NKG2D ligand) and Nectin-2 (DNAM-1 ligand) were higher in EOC patient ascites along with the immune inhibitory cytokines IL-10 and TGF-β1 compared to benign ascites (**Figure 3B**). Besides an increase in inhibitory mediators, markers of immune activation (including IP-10, TRAIL, perforin and IL-12p40 levels) were elevated in malignant ascites samples, soluble factors not shown in **Figure 3B** were not statistically significantly different between malignant and benign reference samples.

**Figure 4:**
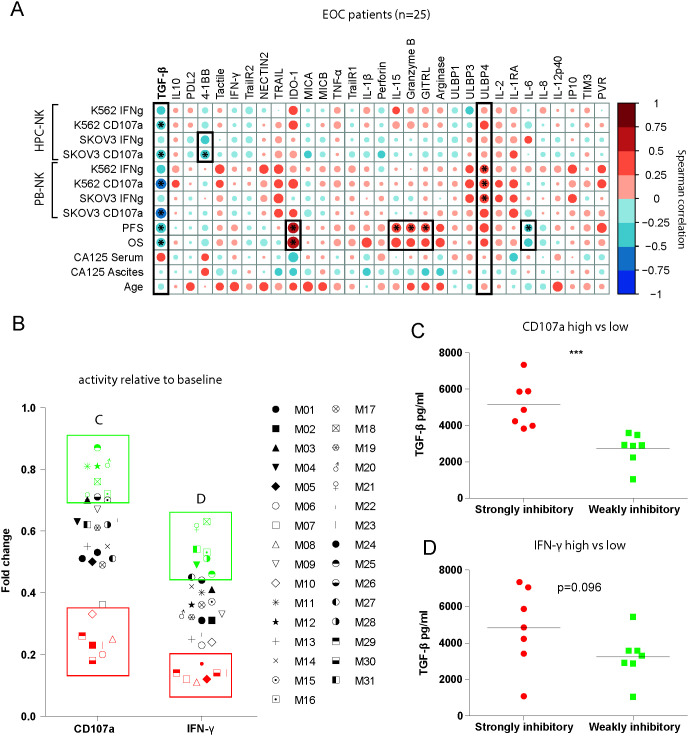
TGF-β1 is a main driver of ascites-induced NK cell inhibition. **(A)** Spearman correlogram of mean fold change of suppression by EOC patient ascites, PFS, OS, CA-125 levels in serum and peritoneal fluids and age versus soluble cytokine levels. A heat map is used to indicate the ρ^2^ of associations between biomarkers. Red indicates a positive correlation, and blue indicates a negative correlation. **(B)** Mean fold change CD107a and IFN-γ inhibition of all conditions for each EOC patient ascites. All red labelled patients indicate the 25% strongest inhibitory ascites’, and all green labelled patients indicate the 25% least inhibitory ascites’. **(C, D)** TGF-β1 levels of most and least inhibitory ascites’ defined in **(B)** based on CD107a **(C)** and IFN-γ **(D)**. A Mann-Whitney test was used for statistical analysis, * p < 0.05, *** p < 0.001.

To define patterns of co-existence for the different soluble mediators in ascites of advanced EOC patients, we correlated their concentrations (**Figure 3C**). Most mediators were positively correlated, indicating that soluble factor levels accumulate in a joint fashion with 19 soluble factors strongly positively correlating together and to a lesser extent with the remaining 10 soluble factors. Notably, TGF-β1 was not part of this cluster, as correlations with other determined mediators were lacking, and only IDO-1 was negatively correlated. Next, we assessed correlations between ascites-induced NK cell dysfunction and clinical parameters with the levels of determined soluble factors in ascites in our patient cohort (**Figure 4A**). Interestingly, the presence of high peritoneal TGF-β1 concentrations correlated to a decreased NK cell functionality. Soluble 4-1BB exhibited a similar, but weaker, correlation with ascites-mediated NK cell dysfunction (**Figure 4A**). Conversely, high levels of ULBP-4 were positively correlated with NK cell-mediated EOC reactivity, although, only in 9 out of 31 tested ascites samples the ULBP-4 concentration was above the limit of detection (data not shown). When focusing on associations with clinical parameters, we observed that IL-15, GITRL and granzyme-B were positively correlated to PFS. Surprisingly, IDO-1 also positively correlated to PFS and OS (**Figure 4A**). In contrast, TGF-β1 and IL-6 negatively correlated with PFS, and TGF-β1 negatively associated with OS as well. None of the assessed cytokines were significantly correlated to serum and peritoneal levels of CA-125 (**Figure 4A**). Finally, we analyzed the most and least NK cell inhibitory ascites (defined by quartiles of CD107a and IFN-y activity relative to the baseline functionality) for their TGF-β1 concentration (**Figures 4B-D**). This confirmed that TGF-β1 was significantly increased in the strongest NK cell inhibitory EOC ascites samples. Taken together, these data suggest that TGF-β1 is a soluble factor that strongly negatively correlates to ascites-induced NK-cell dysfunction and decreased survival in EOC patients.

### 3.3 TGF-β1 inhibits HPC-NK cell proliferation and functionality

**Figure 5:**
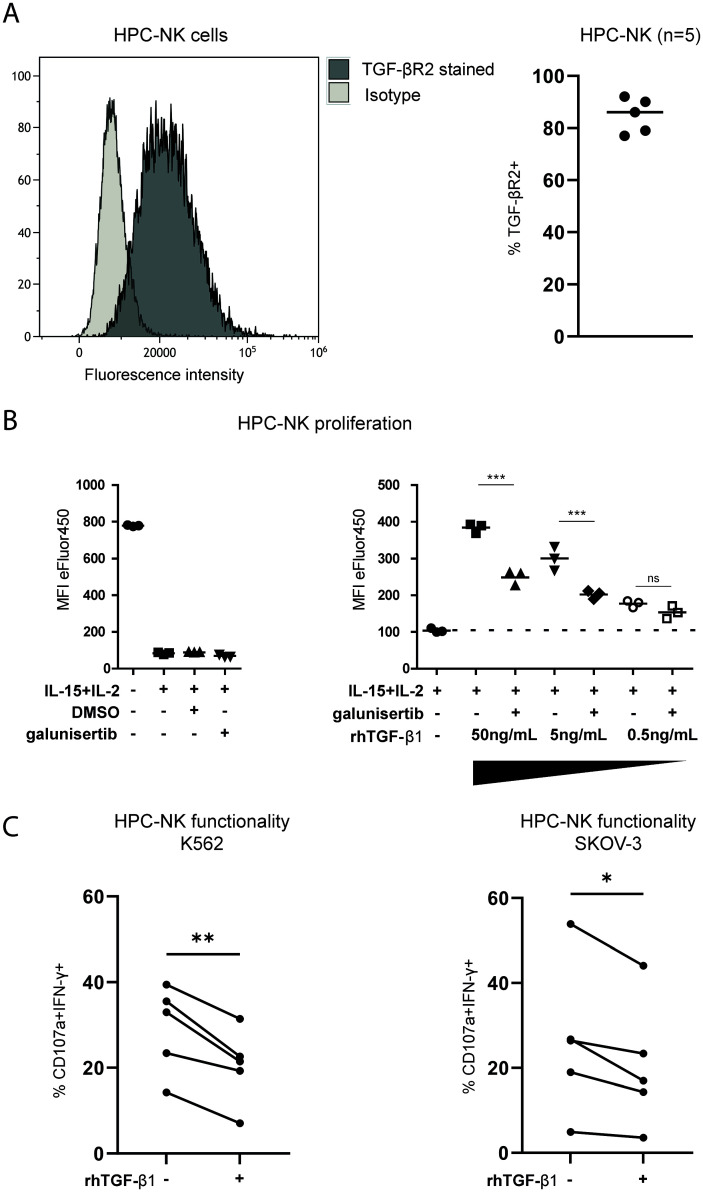
TGF-β1 inhibits NK cell functionality that can be partially rescued by a TGF-β1 small molecule inhibitor. **(A)** TGF-βR2 expression (black) and isotype control (light grey) unstimulated HPC-NK cells in histograms (left) and the percentage of TGF-βR2+ HPC-NK cells shown for 5 different donors (right). **(B)** Cell Proliferation Dye-stained HPC-NK cells in a proliferation assay measured by a decrease in eFluor450 fluorescence. The left panel shows triplicate unstimulated, IL-2+IL-15 stimulated (with or without DMSO or galunisertib) controls with eFluor450 MFI on y-axis. The right panels shows reference triplicates of HPC-NK cells stimulated with IL-2+IL-15 control with or without decreasing amounts (50, 5 or 0.5ng/mL) of rhTGF-β1 with or without galunisertib. Both panels include data from the same experiment. The Friedman test was used to calculate statistical significance. **(C)** Percentage of HPC-NK cells that are double positive for CD107a and IFN-γ after stimulation with K562 or SKOV-3 cells in the presence or absence of TGF-β1(n=5). One-way ANOVA with *post-hoc* Bonferroni test was used for statistical analysis. ns, not significant, *p<0.05, **p<0.01, ***p<0.001.

As a distinct role of TGF-β1 in mediating EOC ascites-induced NK-cell dysfunction was found in our association studies, we further wanted to elucidate the effects of TGF-β1 and its interference on modulating NK-cell EOC reactivity. First, we assessed expression of TGF-βR2, which is part of the heterotetrametric receptor complex, on the surface of HPC-NK cells and observed that TGF-βR2 was highly expressed by HPC-NK cells (**Figure 5A**). Next, we evaluated to what extent rhTGF-β1 inhibits HPC-NK cell proliferation induced by IL-2/IL-15 stimulation and if galunisertib (LY2157299), an affordable and readily available TGF-βR1 kinase inhibitor, can interfere with TGF-β1-mediated effects of proliferation. Indeed, TGF-β1 reduced HPC-NK cell proliferation that could partly be restored by galunisertib (**Figure 5B**). In addition, pre-treatment with rhTGF-β1 significantly impaired HPC-NK cell-mediated IFN-γ production within the CD107a degranulating population upon target cell challenge (**Figure 5C**). Interestingly, galunisertib partially restored rhTGF-β1-induced reduction of IFN-γ responses against K562 and/or SKOV-3 target cells in 4 out of 5 donors (**Supplementary Figure 3**). These data indicate that TGF-β1 could severely hamper functionality of therapeutic NK-cell products and that blockade of TGF-βR1 receptor signaling can partly rescue NK cell dysfunction by TGF-β1.

### 3.4 M2-like TAMs, B cells and Tregs are associated with the secretome including TGF-β1 in EOC patient ascites

**Figure 6:**
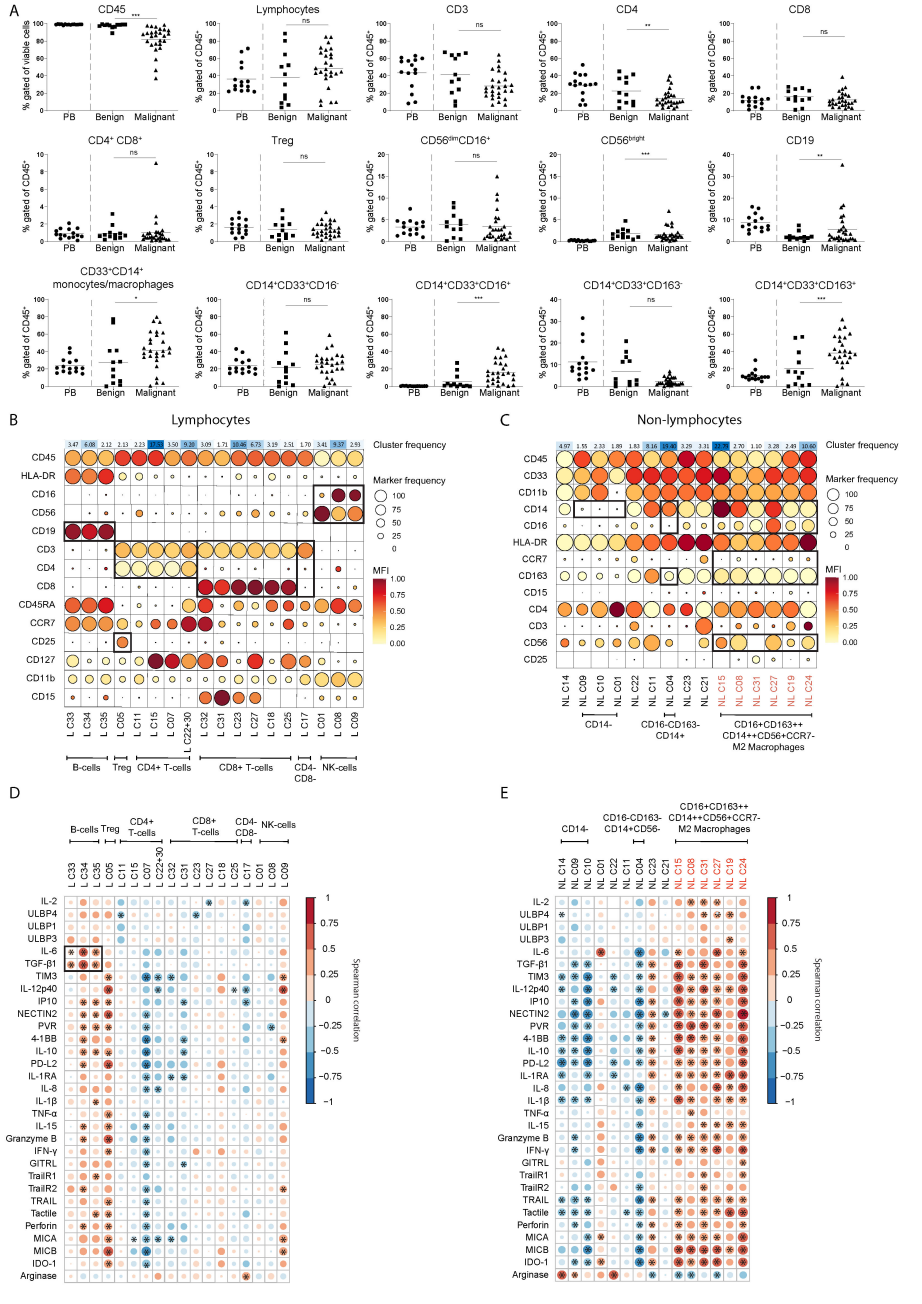
M2 like macrophages, Tregs and B cells are associated to the secretome including TGF-β1 in EOC patient ascites. **(A)** Frequency of cell populations within CD45^+^ cells in PBMCs (as circulating reference, PB), benign peritoneal fluids and malignant ascites based on manual gating, Kruskal-Wallis with Dunn’s Multiple Comparison Test was used for statistical analysis, ** p < 0.01, *** p < 0.001. **(B, C)** Scaled MFI values of FlowSOM clusters for the lymphocyte **(B)** and non-lymphocyte **(C)** fraction of malignant ascites in a balloon plot. Each row represents a marker, while columns represent a cluster. Balloon size represents frequency while balloon color represents MFI (red is high MFI, yellow is low MFI). On top of the balloon plot the cluster size is presented as percent of total where red indicate large clusters and green small clusters. All clusters smaller than 1% of the total cell population were excluded to prevent overfitting of rare populations. **(D, E)** Spearman correlogram of cytokine levels versus clusters of the lymphocyte **(D)** and non-lymphocyte **(E)** fraction of EOC patient ascites. A heat map is used to indicate the Spearman’s rank correlation coefficient (ρ) of associations between biomarkers. Red indicates a positive correlation, and blue indicates a negative correlation. All clusters smaller than 1% of the total cell population were excluded to prevent overfitting of rare populations. ns, not significant, * p < 0.05, L, lymphocyte and NL, non-lymphocyte.

Following the soluble factor analysis, we focused on the cellular compartment in the same cohort of EOC patient and benign reference fluids. For this, we set up an 18-color flow cytometry panel (**Supplementary Table 4**) to assess the immune composition (including T cell subsets, B cells, NK cells, macrophages, monocytic subsets and granulocytic cells) within the peritoneal fluids. PBMC from healthy donors were used as reference in peripheral blood. In benign peritoneal fluids, the fraction of CD45+ hematopoietic cells was 97 ± 3%, while this was 82 ± 15% in ascites of EOC patients, indicating the presence of tumor cells or other non-hematological cells in EOC patient ascites (**Figure 6A**). Notably, the percentage of CD14+CD33+CD16+ and CD14+CD33+CD163+ macrophages in EOC patient ascites was significantly higher (16.3 ± 13.0% and 38.4 ± 20.1%, respectively) compared to the percentages found in benign fluids (5.7 ± 8.8% and 20.3 ± 20.5%, respectively). No significant differences were found in the percentages of classical CD14+CD33+CD16- or CD14+CD33+CD163- monocytes, which was 25.1 ± 13.7% and 2.2 ± 1.8% respectively in EOC patients compared with 21.8 ± 19.9% and 7.0 ± 7.2% in benign fluids. Furthermore, no difference was found in the proportion of lymphocytes within the CD45+ population between EOC and benign fluids, which was 48.9 ± 22.5% and 39.2 ± 29.4%, respectively. The frequency of CD4+ T cells was slightly lower in EOC ascites, while the frequency of CD19+ B cells was slightly higher (**Figure 6A**).

**Figure 7:**
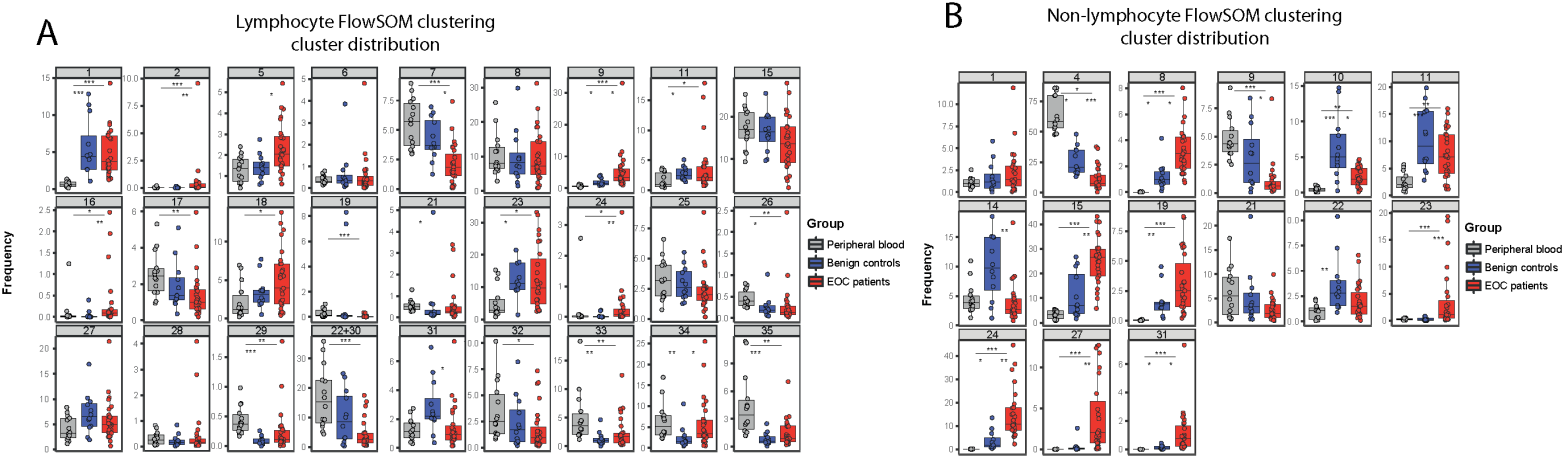
Frequencies of FlowSOM clustering for each individual cluster. **(A, B)** Box and whiskers plots showing the percentage of FlowSOM clusters, based on the lymphocyte fraction **(A)** and non-lymphocyte fraction **(B)**, showing different cell cluster distribution in healthy donors (grey, n=14), benign patients (blue, n=12) and patients with malignancy (red, n=27). All clusters smaller than 1% of the total cell population were excluded to prevent overfitting of rare populations. Kruskal-Wallis with Dunn’s Multiple Comparison Test was used for statistical analysis * p < 0.05, * p < 0.05, ** p < 0.01 and *** p < 0.001.

Next, we performed multidimensional scaling (MDS) on the leukocyte dataset to visualize the differences in distribution of cellular populations in healthy donor PB compared to benign reference or EOC patient peritoneal fluids (**Supplementary Figure 4A**). For the non-lymphocyte (NL) fraction we gated on CD45+CD33+ cells and for the lymphocyte (L) on CD45+CD33- cells, and the MFI of each marker was used to calculate the MDS plot. Healthy donor PB samples clearly clustered separately from EOC patient ascites samples, while benign samples are distributed between the EOC patient ascites samples and healthy donor PB samples. For the CD45+ NL fraction of the leukocyte panel, we found a similar pattern but the differences between each group were less pronounced. Hierarchical clustering of surface marker intensities across the samples corresponded with results from the MDS, showing a clear difference between benign reference and EOC patients which was especially clear in the NL fraction (**Supplementary Figure 4B**). To further define the cell population landscape in ascites, we used the FlowSOM (k=35) clustering approach. We found separate clusters for both the L and NL fractions, and excluded all clusters smaller than 1% of the total cell population to prevent overfitting of rare populations (**Figures 6B, C**). For the L fraction, we found three clusters that were enriched in EOC patients, namely CD4+CD25++CD127- Tregs (L C05), CD56dimCD16low NK cells (L C09) and a cluster of CD19+ B cells (L C34) (**Figures 6B**, **7A**). The two clusters enriched in benign peritoneal fluid were a CD3+CD4+ T cell cluster (L C07) and a CD3+CD8+ T cell cluster (L C31). For the NL fraction, we found a large meta-cluster of CD14+CD163+CD197-CD16+/-CD56+/- M2-like macrophages (NL C08, NL C23, NL C31, NL C33, NL C34, NL C30, NL C26 and NL C29, **Figures 6C**, **7B**). To determine which of these cell populations were associated with the secretory compartment in EOC ascites, we correlated the FlowSOM clustering data with the soluble factor levels. Interestingly, the TGF-β1 and IL-6 concentration was significantly associated with all CD19+ B-cell clusters (L C33, L C34 and L C35, **Figure 6D**). In addition, these CD19+ B cell clusters also associated with other inhibitory and activating factors, including IL-10 and soluble ligands for DNAM-1 (NECTIN2 and PVR). This association was partially shared with the CD4+CD25++CD127- Treg cluster (L C05) and a CD56dim NK cell cluster (L C09) with reduced CD16 expression typically found in EOC ascites (23), although not all correlations were similar. Notably, the strongest association with the secretory milieu in ascites was with CD14+CD163+CD197-CD16+/-CD56+/- M2-like macrophages including TGF-β1 (NL C08, NL C15, NL C19, NL C24, NL C27 and NL C31, **Figure 6E**). A cluster of (classical) CD14+CD163-CD16-CD56- monocytes, found mostly in PB (NL C04), was negatively correlated with several soluble factors.

Altogether these findings indicate that the presence of CD14+CD163+CD197-CD16+/-CD56+/- M2-like macrophages encompasses the major difference between the cellular make-up of benign versus EOC ascites. Interestingly, these M2-like TAMs strongly correlate with the inhibitory secretome of EOC patient ascites, including TGF-β1. Besides these M2-like macrophages, B cells and Tregs are also associated with the secretome found in EOC patient ascites.

## 4 Discussion

EOC patient ascites is a major factor contributing to recurrence and poor prognosis in EOC patients. This ascites contains a variety of soluble and cellular components that influence the function of tumor-targeting lymphocytes (5, 24). Although significant NK-cell numbers are present in EOC ascites, their antitumor activity is inhibited by soluble mediators and membrane-bound receptors (25). Furthermore, it is well known that ascites can impair the functionality of NK cells (5), and can thereby hamper the antitumor efficacy of NK-cell therapeutic products in EOC patients. Nevertheless, we and others have previously shown that ascites-induced NK-cell dysfunction can be reinvigorated by for instance IL-15 receptor-mediated stimulation (13, 26). To further elucidate mechanisms impairing NK cell function in the local EOC environment, we now combined NK-cell functional data, soluble factor analysis, high-dimensional flow cytometry assessment of cellular components and clinical parameters of advanced EOC patients in integrated analyses. Using an activation assay, we examined the effects of ascites on CD107a and IFN-γ expression of NK cells after stimulation with tumor target cells. Interestingly, we found that strong inhibitory effects of EOC patient ascites on CD107a degranulation from healthy donor-derived NK cells were associated to reduced PFS and OS in a cohort of 31 high-grade serous EOC patients. One explanation why survival was significantly associated to CD107a degranulation but not IFN-γ is that the baseline IFN-γ response to SKOV-3 is low, especially for healthy-donor PB-NK cells thus reducing demonstration of the inhibitory potential of ascites. Further, for most ascites fluids maximum IFN-γ inhibition was achieved, and a stronger OC cell stimulus in a future study might overcome this limitation. Moreover, we observed a positive correlation between ascites-induced NK-cell dysfunction and higher CA-125 levels in the serum of the EOC patients. Previous reports have already shown that CA-125 present in EOC patient ascites inhibits NK-cell antitumor activity (27). However, our data show a stronger correlation between ascites-induced NK-cell dysfunction and CA-125 serum levels rather than peritoneal levels. Furthermore, we revealed that TGF-β1 was a suppressive factor associated to ascites-induced NK-cell dysfunction, reduced patient survival and the presence of a higher frequency of M2-like macrophages. In functional assays, we showed that NK cell proliferation and function is significantly affected by TGF-β1, and inhibition of TGF-βR1 signaling by galunisertib partly restored NK-cell functionality. Although we tried to capture the major soluble factors and pathways that are known to impact NK cell function such as IL-15, IL-10 and ligands for activating receptors such as DNAM-1 and NKG2D we did not find a significant association of any other factor with both functional and patient survival data. As TGF-β1 is only partly responsible for our findings there may be important factors that we failed to include in our study or that our study was not power enough to identify. Nonetheless, these results show that TGF-β1 is an important factor in EOC ascites capable of dampening NK-cell antitumor activity.

Other factors that were significantly elevated in EOC patient ascites compared to benign ascites included IL-6 and IL-10 as well as soluble ligands for activating NK cell receptors such as MIC-B (NKG2D ligand) and Nectin-2 (DNAM-1 ligand). High ascites levels of IL-6 have previously been associated with poor outcome in EOC patients (28). For inhibitory cytokine IL-10, 2 out of 3 studies showed that IL-10 levels in ascites were not associated with prognosis (28). Our study did not show an association of IL-10 levels to patient survival either. Shedding of ligands for activating receptors such as MIC-A and MIC-B can help evasion of tumor cells from NK-cell attack (29), and we found that elevated MIC-B levels are increased in EOC ascites compared to benign ascites. Recent data suggests similar mechanisms where soluble PVR, a DNAM-1 ligand, inhibited DNAM-1-mediated NK-cell activation (20). In contrast, we found that soluble ULBP-4 in ascites, a different NKG2D ligand, was associated to increased NK-cell activity similar to a recent study where ULBP4 was associated with higher T-cell function in Multiple Sclerosis patients (30). Although benign ascites samples also showed strong inhibitory effects on NK cells, our sample size was too small to perform comprehensive correlative studies and we may have missed important inhibitory soluble factors in our analysis. The mechanisms underlying these observations will be further studied in follow-up investigations.

Many of the soluble inhibitory factors assessed are not secreted by NK cells, but other immune cells that regulate immunity, including NK cells, in ascites. We also evaluated the frequencies and phenotype of a variety of immune cells in order to identify the cell populations that correlate with the suppressive factors present in ascites. We found a lower fraction CD45+ hematopoietic cells in patients with malignancy, which corresponds to increased frequency of tumor and stroma cells present in ascites (13). Another evident observation was the high proportion of M2-like TAMs, which has also been described and reviewed extensively (31). Presence of these TAMs was associated with high levels of TGF-β, IL-6, IL-10 and TNF-α in ascites (32), which we confirmed in our study. It is well-known that TGF-β is a major driver for the polarization towards M2-like TAMs (33). In addition, we observed that other soluble factors were associated with these TAMs, including IL-12p40, IL-15, IP10 and soluble IDO-1. Since our study is solely associative, future research is required to elucidate the source of these factors. Generally, IDO-1 expression is known to be up-regulated in cancer and serves as an immunosuppressive and immune evasive mechanism by tumor cells (34). A recent study on IDO-1 in EOC demonstrated elevated levels of IDO-1 metabolism in ascites compared to healthy controls (35). In contrast, we did not observe an increase for soluble IDO-1 in our study, but we compared EOC patient ascites to benign reference fluids, while Grobben et al. used healthy donor plasma as controls potentially explaining the discrepancy in results. Furthermore, our study revealed a positive correlation between survival of EOC patients and increased IDO-1 levels. Moreover, in a phase III trial IDO-1 inhibition did not show survival benefits in unresectable or metastatic melanoma patients (36). Therefore, the mechanism behind IDO-1 signaling in cancer remains to be further investigated as IDO-1 has been associated strongly with ascites and survival of EOC patients.

Besides M2-like TAMs we observed associations of (sub)populations of B cells and Tregs and high levels of soluble mediators in EOC patient ascites. Regulatory (CD25+) B cells have already been described, and few studies have highlighted the importance of B cells in EOC (37). One study on regulatory IL-10-producing B cells in EOC patients showed that their frequencies correlated positively with Tregs (38). These IL-10-producing B cells also suppressed IFN-γ production by effector T cells. Furthermore, Tregs are known to be associated to an inhibitory TME. Our study confirms that Tregs in EOC are associated with the secretome, although these Tregs did not significantly correlate to TGF-β1 concentrations they did to IL-10. The high proportion of CD56dimCD16low NK cells in ascites was previously reported to be associated to EOC (23). Although TGF-β1 is known to downregulate NK-cell phenotype and function, we found no correlation between the CD56dimCD16low NK-cell phenotype and TGF-β1.

Others have shown that TGF-β1 can promote cancer progression, which is primarily mediated through its effects on the local TME. Here, we revealed that TGF-β1 in EOC ascites contributes to induction of NK-cell dysfunction. CD34 HPC-derived NK cells were susceptible to rhTGF-β1 added to the culture leading to reduced proliferation and functionality. Importantly, addition of the TGF-βR1 inhibitor galunisertib partly mitigated this inhibitory effect, but this effect was donor dependent. Moreover, while galunisertib was positively evaluated in some (pre-)clinical studies (39, 40), systemic TGF-β inhibition yielded poor results overall that were attributed in part to cardiovascular toxic side effects and formation of benign tumors (33, 41). In combination with our data, this shows that a more effective and controlled inhibition of TGF-β1 signaling is needed for sustained activity of NK cells in the TME. This is currently being explored by others via overexpression of a dominant-negative TGF-βR2 (TGFβRDN) or CRISPR-Cas9-mediated knockout of TGFBR2 in adoptive T- and NK-cell therapy products (42, 43). Recently, a phase I trial with prostate cancer-directed CAR T cells armored with a TGFβRDN to block TGF-β signaling was conducted illustrating both feasibility and importance of interfering with the TGF-β pathway (44). Another study in glioblastoma-bearing mice treated with allogeneic NK cells showed that TGFBR2 knockout prevented NK-cell dysfunction and tumor growth (45). The same group is currently evaluating NK cells with deleted TGF-βR2 for treatment of glioblastoma in a phase I trial (NCT04991870). This CRISPR/Cas9 TGF-βR2 deletion can easily be implemented in our protocol for ex vivo-generated HPC-NK cells. In combination with the aforementioned TGF-β receptor gene editing strategies, this provides a promising approach to improve current EOC treatment with adoptive NK cell therapy.

In this study, we primarily used CD34+ HPC-derived NK cells that have already been shown to be able to efficiently kill ovarian cancer cells *in vitro* and *in vivo* (46), and these cells are currently tested in a phase I study in EOC patients (NCT03539406). Importantly, we revealed that galunisertib significantly rescued HPC-NK cell function when challenged with TGF-β *in vitro*. Furthermore, while both HPC-NK cells and healthy donor PB-NK cells were potently impaired by ascites of EOC patients, we found that HPC-NK cells were more resistant to ascites-mediated suppression indicating their high functionality and lower vulnerability to TME-mediated suppression. This finding is in line with a previous report where we showed that HPC-NK cells have superior EOC reactivity compared to PB-NK cells, which could be related to the increased granzyme-B release by HPC-NK cells and higher serial killing potential (21, 47). Furthermore, we found TGF-βR2 expression to be higher on PB-NK cells than on HPC-NK cells (data not shown).

In conclusion, our results demonstrate that high peritoneal TGF-β1 concentrations were negatively correlated to EOC patient survival. Furthermore, we found that NK-cell function is strongly impaired by ascites from high-grade EOC patients, and that TGF-β1 plays an important role in this effect. We showed that inhibition of the TGF-β signaling pathway by small molecule inhibitor galunisertib partly rescued NK-cell function. Taken together, these results provide a rationale for improvement of NK-cell function via inhibition of TGF-β1 signaling. Since systemic anti-TGF-β has shown limited success in clinical trials so far, a more potent approach with fewer side effects is warranted. We envision implementing CRISPR/Cas9 gene editing in our 5-week culture protocol to improve our HPC-NK cell therapeutic product to achieve this.

## Data Availability

The raw data supporting the conclusions of this article will be made available by the authors, without undue reservation.
