# Supplementary figures and images for: Increased peritoneal TGF-β1 is associated with ascites-induced NK-cell dysfunction and reduced survival in high-grade epithelial ovarian cancer
Source: Front Immunol. 2024 Sep 23;15:1448041. doi: 10.3389/fimmu.2024.1448041 (PMC11456434; doi:10.3389/fimmu.2024.1448041)

FIGURE S1

16h ascites treatment

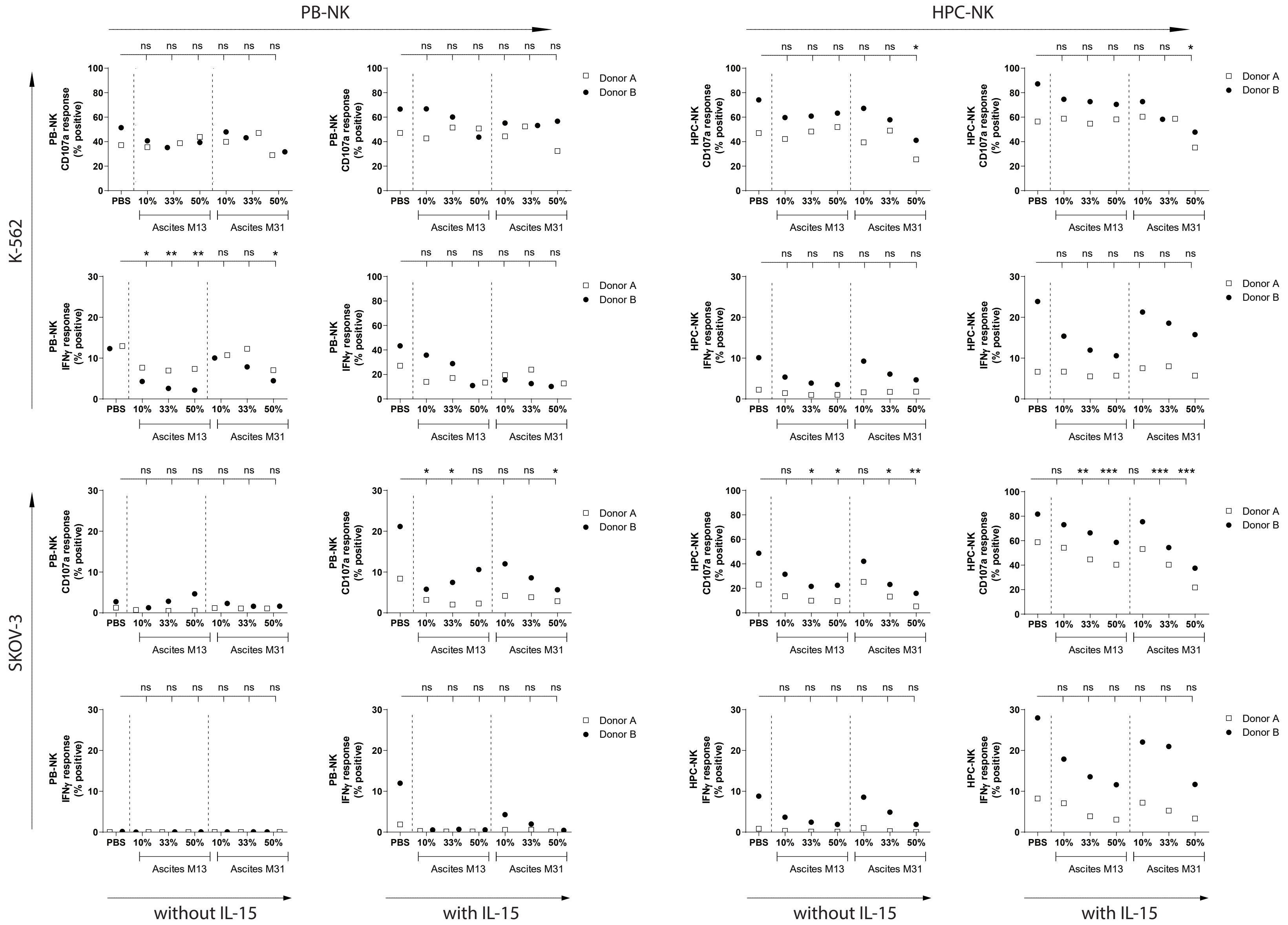

Supplement: Supplementary file 1 [file DataSheet1.pdf]

FIGURE S2

4h ascites treatment

K-562

SKOV-3

PB-NK

HPC-NK

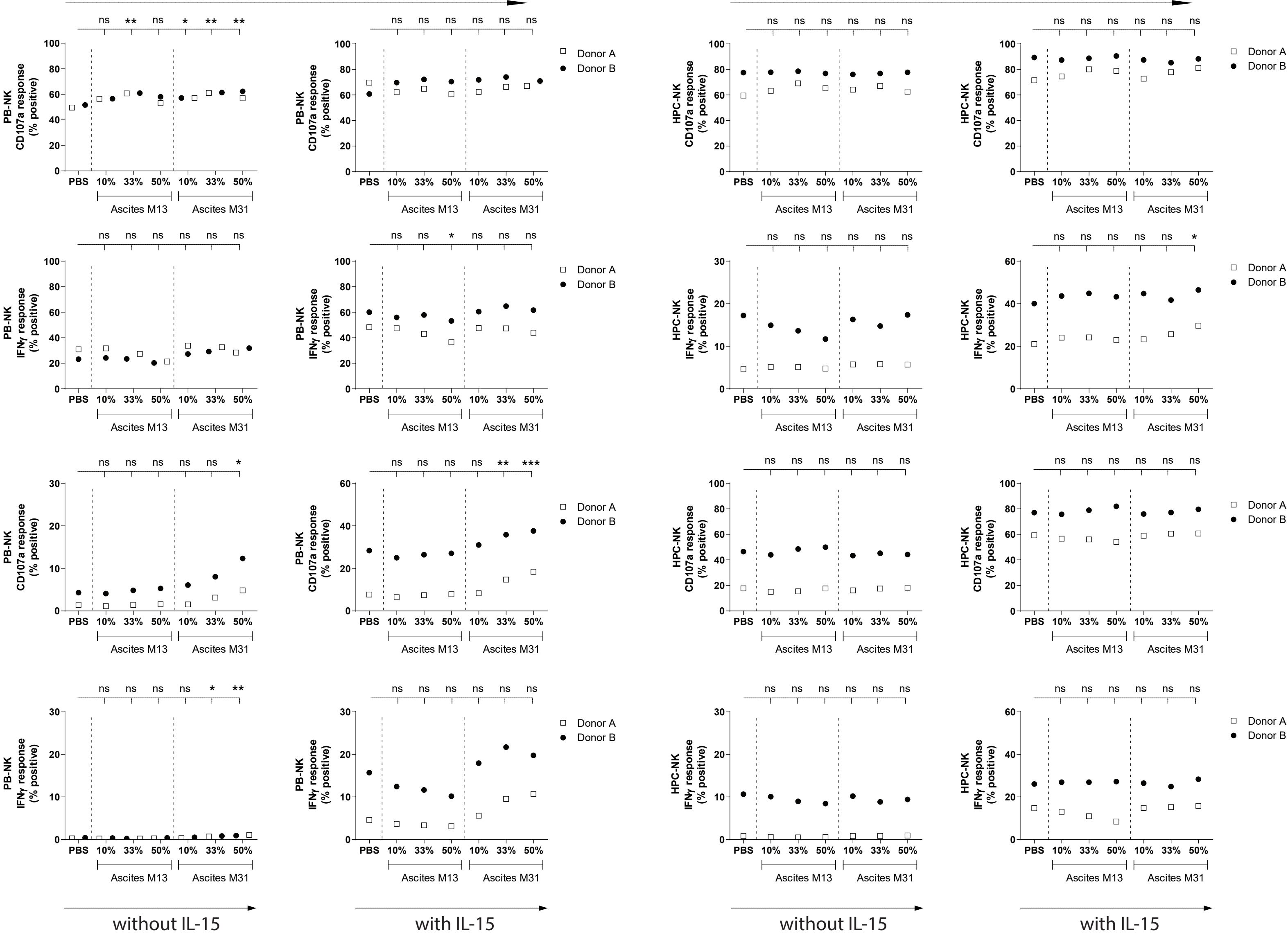

Supplement: Supplementary file 2 [file DataSheet2.pdf]

A **FIGURE S3**

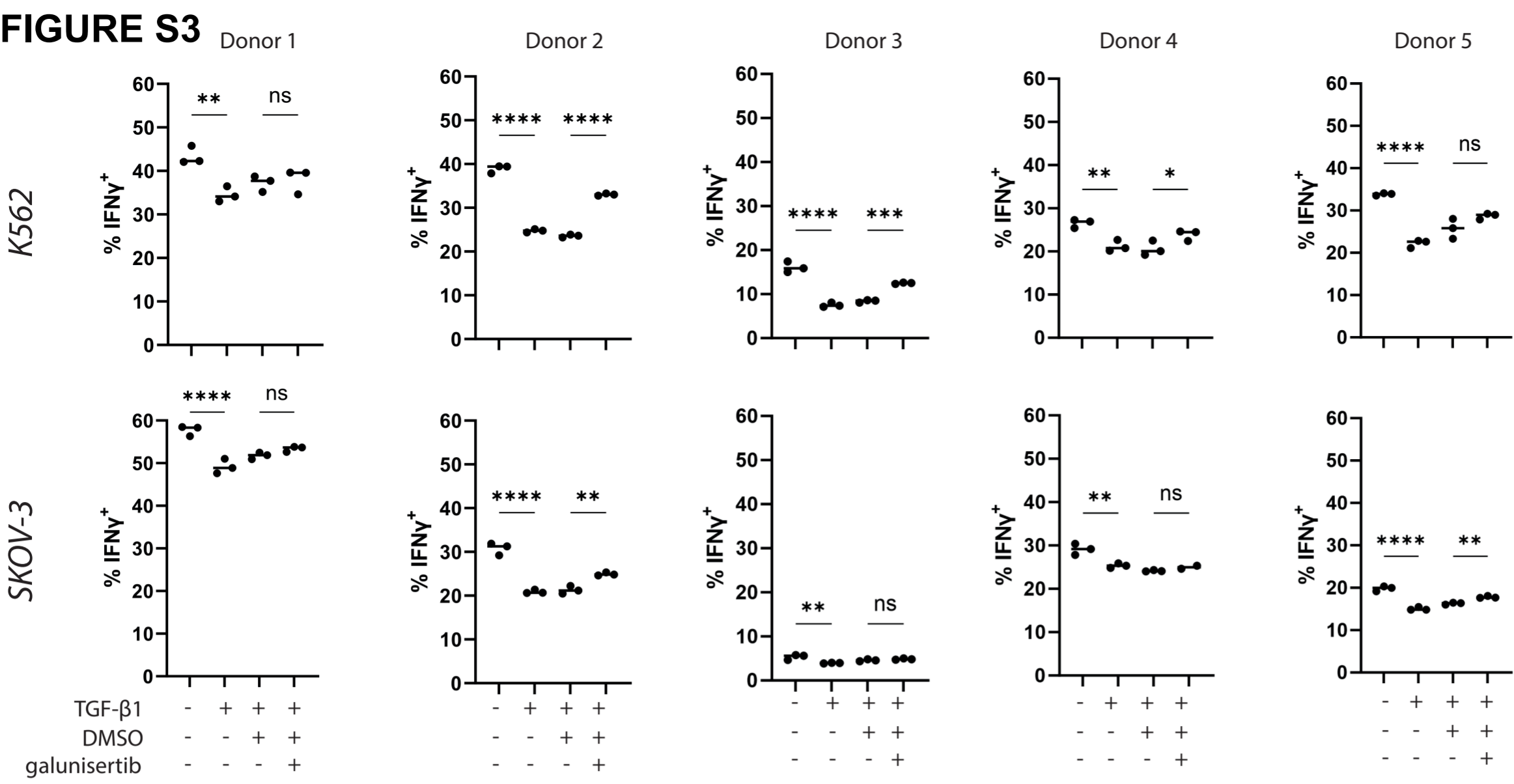

B

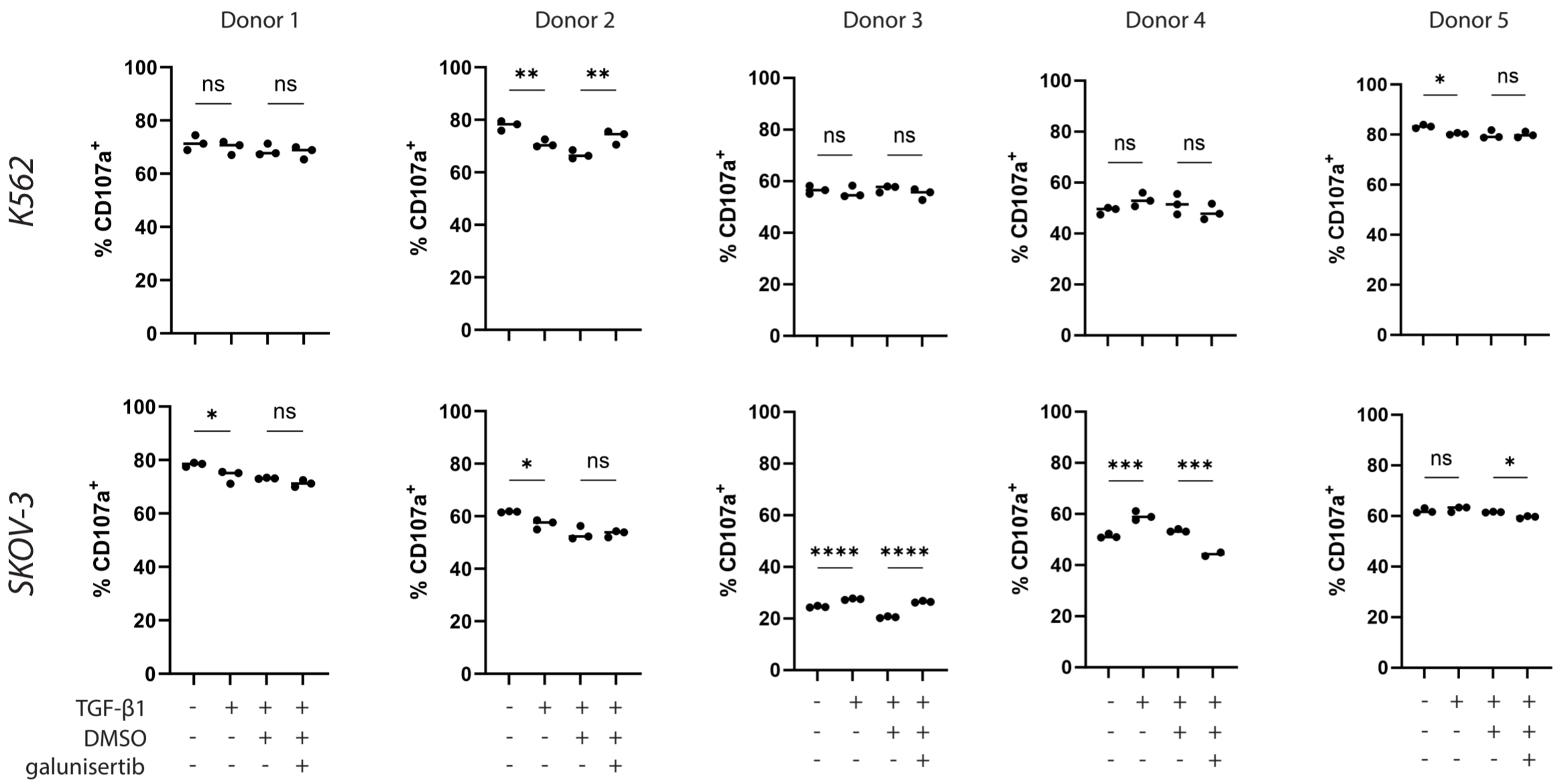

Supplement: Supplementary file 3 [file DataSheet3.pdf]

FIGURE S5

Healthy donor

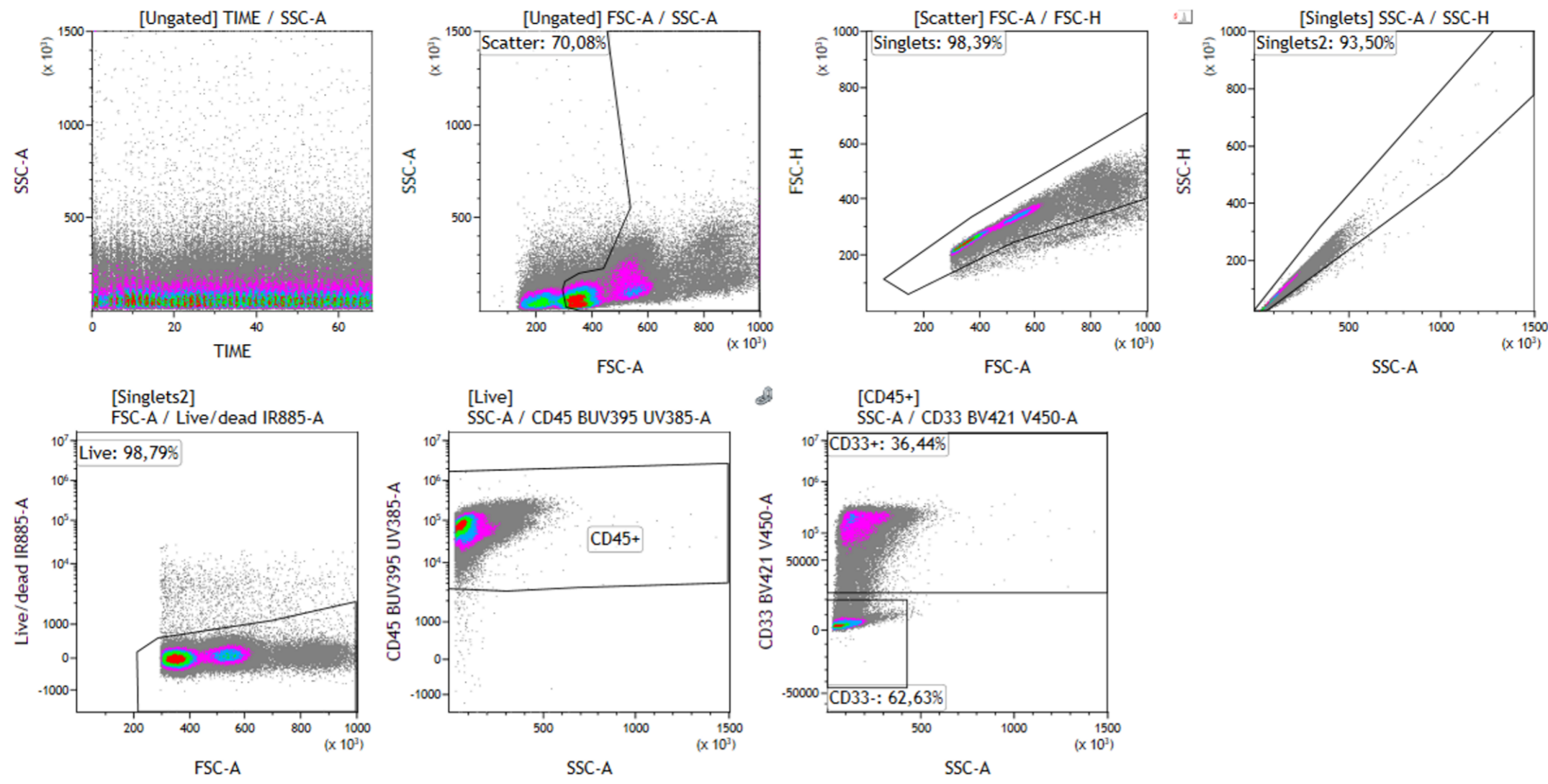

Benign reference

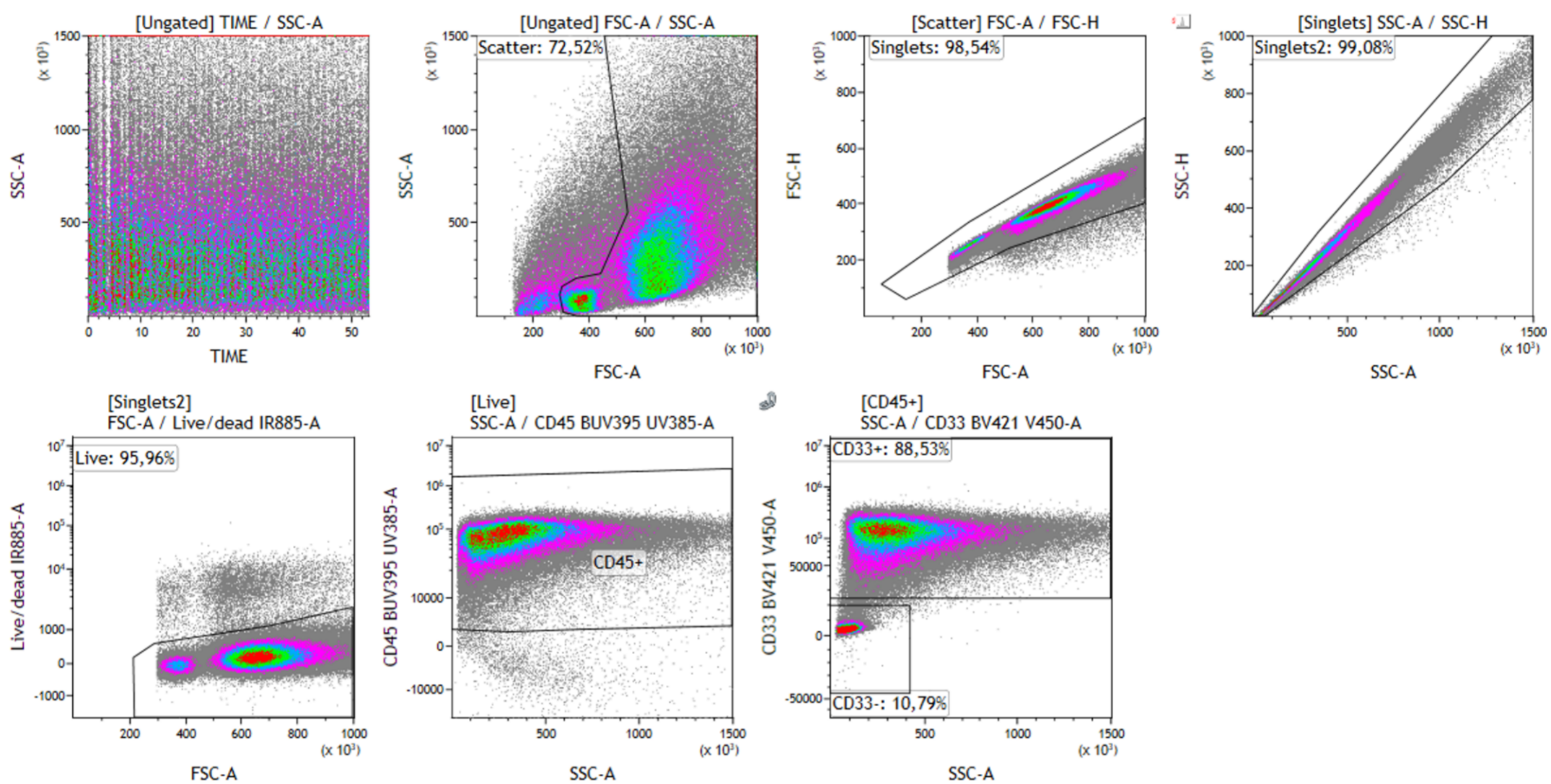

EOC patient

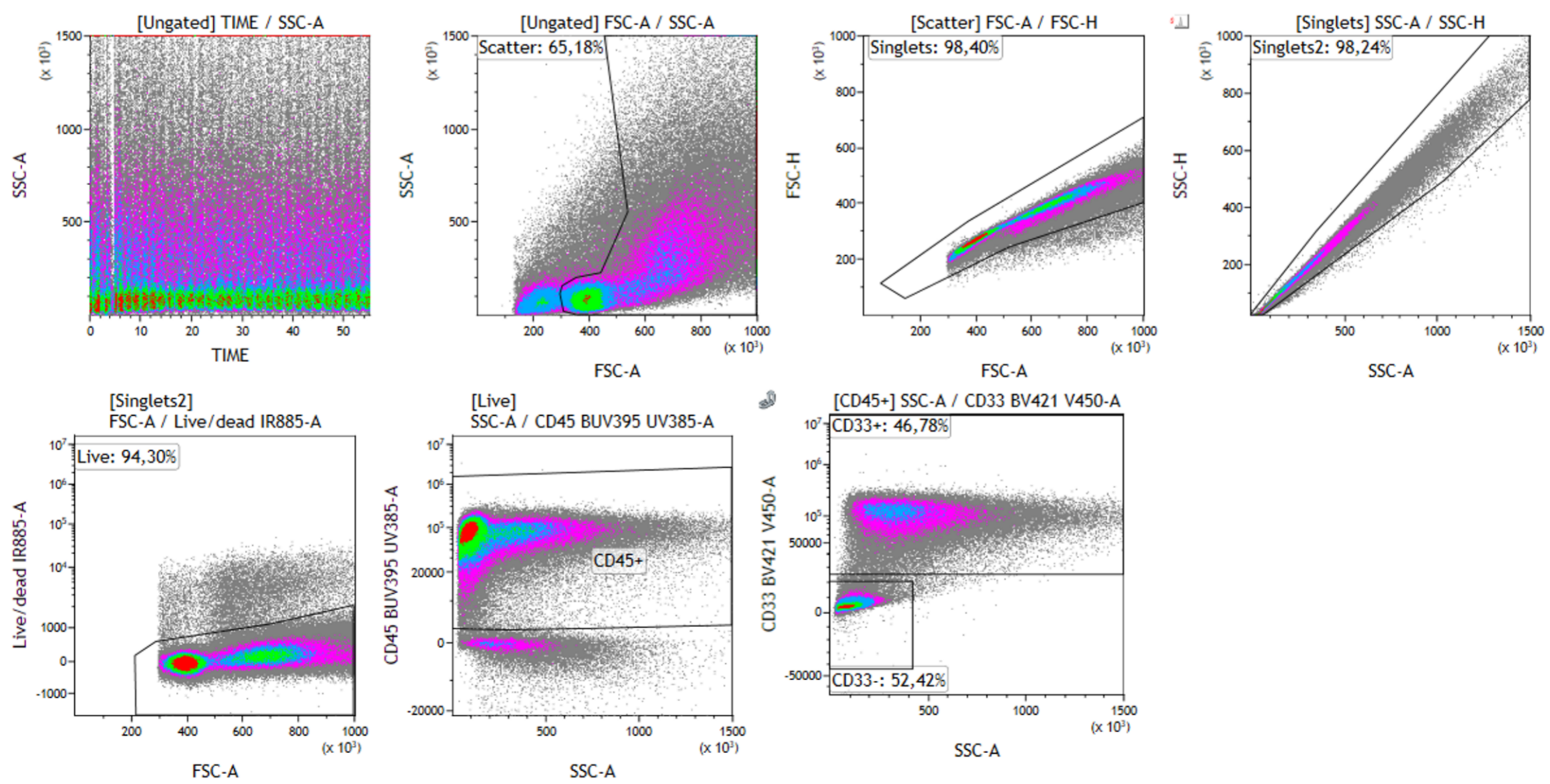

Supplement: Supplementary file 5 [file DataSheet5.pdf]
