# Supplementary material for: Increased peritoneal TGF-β1 is associated with ascites-induced NK-cell dysfunction and reduced survival in high-grade epithelial ovarian cancer
Source: Front Immunol. 2024 Sep 23;15:1448041. doi: 10.3389/fimmu.2024.1448041 (PMC11456434; doi:10.3389/fimmu.2024.1448041)
Supplement: Supplementary file 4 [file DataSheet4.pdf]

FIGURE S4

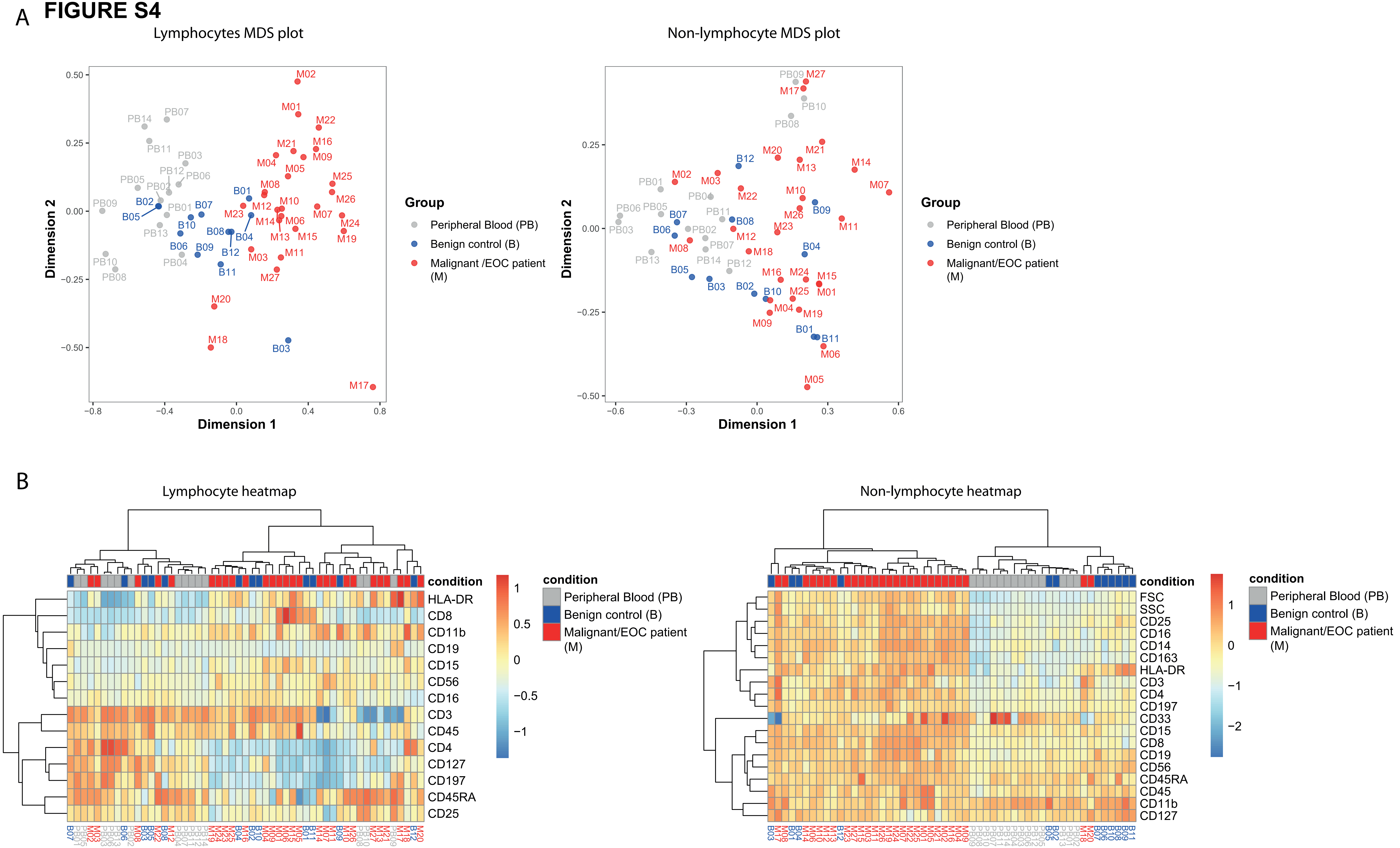

A
Lymphocytes MDS plot
Non-lymphocyte MDS plot
Dimension 1
Dimension 2
Group
Peripheral Blood (PB)
Benign control (B)
Malignant /EOC patient (M)
B
Lymphocyte heatmap
Non-lymphocyte heatmap
condition
Peripheral Blood (PB)
Benign control (B)
Malignant/EOC patient (M)
HLA-DR
CD8
CD11b
CD19
CD15
CD56
CD16
CD3
CD45
CD4
CD127
CD197
CD45RA
CD25
FSC
SSC
CD14
CD163
CD33
