# Supplementary material for: Increased peritoneal TGF-β1 is associated with ascites-induced NK-cell dysfunction and reduced survival in high-grade epithelial ovarian cancer
Source: Front Immunol. 2024 Sep 23;15:1448041. doi: 10.3389/fimmu.2024.1448041 (PMC11456434; doi:10.3389/fimmu.2024.1448041)
Supplement: Supplementary file 6 [file DataSheet6.pdf]

Supplementary information

Supplementary materials and methods

Determination of soluble factors in ascites and serum

Ascites of EOC and benign patients was assessed for soluble TIM-3, GITRL, MICA, Trail-R2, Trail-R1, MICB, IP-10, CD137/4-1BB, IL-10, Nectin-2, Tactile, IFN-γ, TNF-α, IDO, Arginase, Perforin, PVR, IL-12p40, IL-15, TRAIL, PD-L2, Granzyme-B, ULBP-1, ULBP-3 and ULBP-4 using Luminex (ProcartaPlex Thermo Fisher), according to the manufacturers' instructions. Concentrations of human IL-1β, IL-1RA, IL-6, IL-8, TGF-β1 (R&D systems) and IL-2 (431801, Biolegend) were determined using specific commercial ELISA kits, according to the manufacturer's instructions. CA-125 levels were measured at diagnosis by an electrochemiluminescence immunoassay on a random access analyser (Elecsys CA 125 II, Cobas E801, Roche Diagnostics, Germany). Ascites was measured in a 1:50 dilution with Diluent Universal (Roche).

Flow cytometry

For phenotypical analysis, 1 million ascites cells were prepared in 50 µL human serum albumin (HSA) buffer (PBS/0.5% HSA, Sanquin Bloodbank) containing 0.1 mg/ml nanogam (human immunoglobulins, Sanquin Bloodbank). Antibody mix was prepared in a total volume of 55 µL brilliant stain buffer (BD Bioscience, 563794) and antibodies (see Supplemental Table 4). The antibody mix was spun at 14.000g for 2 min. Then, 50 µL antibody mixture was taken, added to the cell suspension and incubated at 4°C for 30 min. Cells were washed twice with 500 µL HSA buffer and resuspended in 100µL HSA buffer for acquisition on a Cytoflex LX flow cytometer (Beckman Coulter). The data was analysed with Kaluza 2.1 and Flowjo v10.

TGF-βR2 expression on NK cells was assessed on overnight cultured NK cells in IMDM supplemented with 10% FCS with or without 1 nM rhIL-15. The next day, cells were harvested, washed and stained for 20 min at 4°C with anti-human TGF-β Receptor II (BioLegend, 399704) and washed again. Dead cells

were excluded using Fixable Viability Dye eFluor780 and samples were acquired on the Cytoflex flow cytometer / analysed with Kaluza software (version 2.1).

High-dimensional flow cytometry data analysis

Flow Cytometry Standard (FCS) 3.0 files were imported into Kaluza 2.1, and analysed by standard gating to remove duplicates and dead cells. Subsequently, the relevant cell populations ($CD45^{+}CD33^{-}CD14^{-}$ lymphocytes and $CD45^{+}CD33^{+}$ non-lymphocytes) were identified (Supplemental Figure 5). These populations were exported to CSV files and imported in R (version 4.0.1), whereupon the data was analysed as described by Brummelman et al [22]. In summary, data was arcsinh transformed using the transFlowVS function of the flowVS package (version 1.19.0) and scaled and centerred using the scale function. Multi-dimensional scaling (MDS) plots, based on the median expression values of each marker, were generated using the plotMDS function from the limma package (version 3.44.3). Next, samples were downsampled (10,000 cells for each sample), concatenated and cell subpopulations were identified using the FlowSOM algorithm (version 1.20.0) [23]. For the lymphocyte fraction CD3, CD11b, CD19, CD45RA, CD127, HLA-DR, CD15, CD4, CD197, CD56, CD25, CD45, CD16 and CD8 were used for FlowSOM clustering and for the non-lymphocyte fraction this was CD3, CD14, CD11b, CD163, CD19, CD45RA, CD127, HLA-DR, CD15, CD33, CD4, CD197, CD56, CD25, CD45, CD16 and CD8. The optimal number of metaclusters were k=35 for both lymphocytes and non-lymphocytes. All clusters smaller than 1% of the total cell population were excluded. Heatmaps were made using the pheatmap package (version 1.0.12) in which hierachical clustering was performed using the Euclidean distance and Ward-linkage. Additional figures were made using the ggplot2 package (version 3.3.2). Subsequently, the cluster data were embedded in the FCS files, which were further analysed in FlowJo software (version 10) and Kaluza (version 2.1) to determine the frequency of positive cells for each marker and the corresponding Mean Fluorescent intensity (MFI). The balloon plot, showing the frequencies and MFI of each marker per cluster, was generated in R using the ggpubr package (version 0.4.0). Heatmap was generated using the Tercen data analytics platform. Data was imported with CSV files and log scaled. The limit of detection (LOD) value was used for all samples at or beyond LOD.

NK cell functionality assays

Potency assay - TGFβ1 pretreatment

Cryopreserved $CD34^+$ progenitor-derived HPC-NK cells were thawed and cultured in the presence of IL-15 and IL-12 as described above. After one week, 100,000 cells were plated per well in a 96-well flat bottom plate in IMDM supplemented with 2% HS and 5 ng/ml IL-15. Cells were cultured for 24 hours in the presence or absence of TGF-β1 (10 ng/ml; Immunotools, 11343160), galunisertib (20 µM) or DMSO (control). After 24 hours, HPC-NK cells were challenged with 100,000 K562 or SKOV-3 cells in the presence of CD107a-PE/Cy7 (clone H4A3, Biolegend, #328618) and Brefeldin A (1 µl/ml; BD Biosciences, #555029). After incubating for 4 hours at 37 °C, cells were harvested and washed with PBS. Cells were stained with eFluor780 (eBiosciences, #65-0865-14), CD45-AF700 (clone HI30, BioLegend, #304024), and CD56-APC (clone HCD56, Biolegend, #318310) before fixation and permeabilization according to the kit's manufacturer's instructions (eBiosciences, #00552300). Finally, cells were stained intracellularly with IFN-y-FITC (clone B27, BD Bioscience, #554700) and Perforin-PE (clone dG9, BioLegend, #308106) before acquisition on Gallios or Cytoflex (Beckman Coulter).

Potency assay – Ascites pretreatment

$CD34^+$ progenitor-derived HPC-NK cells were plated in a flat bottom 96-well plate (Corning Star) at 100,000 cells/well and cultured overnight in IMDM supplemented with 10% FCS with or without 1 nM rhIL-15 (Immunotools) and 100 µL cell free ascites fluid or PBS (control). After overnight incubation, NK cells were challenged with SKOV-3 or K562 and incubated/processed similarly as described above.

Proliferation assay

For the proliferation assay, HPC-NK cells were labeled with eFluor450 (eBioScience, 65-0842-85) and cultured in IMDM supplemented with 10% FCS with or without rhIL2 (1000 U/mL, Chiron, NDC 53905-991-01) and rhIL-15 (20 ng/ml). Cytokines were refreshed on day 3 and FCM analysis was performed on day 7. Dead cells were excluded using Fixable Viability Dye eFluor780 (eBiosciences, 65-0865-18).

Supplementary figure legends

Supplemental figure 1: The effect of ascites from two OC patients with overnight co-incubation on NK cell activity

Percent positive CD107a and IFN-γ on HPC-derived (right) or PB-NK cells (left) stimulated with SKOV-3 (bottom) or K562 (top) target cells in the presence of PBS control or malignant ascites (M13 and M31). Cells were incubated overnight with aforementioned fluids with or without addition of 1nM rhIL-15. After overnight incubation NK cells were challenged with target cells for 4h. Experiment was performed in duplicate, averages are shown.

Supplemental figure 2: The effect of ascites from two OC patients with 4h co-incubation on NK cell activity

Percent positive CD107a and IFN-γ on HPC-derived (right) or PB-NK cells (left) stimulated with SKOV-3 (bottom) or K562 (top) target cells in the presence of PBS control or malignant ascites (M13 and M31). Cells were incubated for 4h with aforementioned fluids with or without addition of 1nM rhIL-15 and target cells. Experiment was performed in duplicate, averages are shown.

Supplemental figure 3: TGF-β1 inhibits NK cell IFN-γ responses and can be rescued by a TGF-β1 small molecule inhibitor in a subset of donors

*Percent positive IFN-γ **(A)** and CD107a **(B)** positive of five different HPC-derived NK cell donors stimulated with SKOV-3 (bottom) or K562 (top) target cells in the presence or absence of rhTGF-β1, DMSO and galunisertib. Experiment was performed in triplicate.*

Supplemental figure 4: PCA clustering and hierarchical clustering of healthy donors, benign patients and malignant patients

***(A)** Principal component analysis (PCA), based on the lymphocyte distribution (left) and non-lymphocyte population (right), showing (dis)similarities in healthy donors (grey, n=14), benign patients (blue, n=12) and patients with malignancy (red, n=27) are shown. **(B)** Scaled expression levels in healthy donors (n=14) of Malignant (n=26) and benign (n=12) visualized in a heatmap. Each row represents a different*

expression marker, while columns represent patients or donors (H=healthy donor, M=malignant and B=benign). The scaled expression levels are reported and visualized with a color scale from blue (low levels) to red (high levels).

Supplemental figure 5: Frequencies of FlowSOM clustering for each individual cluster

Representative flow cytometry gating strategy of a healthy donor (top), benign (middle) and EOC patient (bottom) is shown as an example to demonstrate the gating strategy used to gate CD45+CD33+ and CD45+CD33- cells for the FlowSOM analysis. First we gated on a steady cell input using a time gate. Then debris was excluded based on forward scatter (FSC-A) vs. side scatter (SSC-A). Doublets were excluded by plotting FSC-Area vs FSC-Height and SSC-Area vs. SSC-Height. Dead cells were excluded with a viability dye. CD45+ cells were displayed and gated vs. SSC. CD33+ and CD33- cells were then defined based on CD33 expression.

Supplementary tables

Supplemental Table 1. Overview patient characteristics.

		Malignant (n=31)	Benign (n=16)
	**Mean age (SD)**	63 (11) years	53 (10) years*
	**Median PFS (SD)**	6.9 (8.9) months	N/A
	**Median OS (SD)**	18.9 (14.9) months	N/A
	**Serum CA-125 (SD)**	2432 (2879) U/mL	244 (636) U/mL**
	**Peritoneal CA-125 (SD)**	17589 (23098) U/mL	1026 (1891) U/mL
	**FIGO stage 3b/c**	n=18	N/A
	**FIGO stage 4**	n=13	N/A
	**Alive*****	n=7	N/A
**Treatment type**	**NACT**	n=21	N/A
	**PDS**	n=7	N/A
	**No treatment**	n=2	N/A
**Response**	**Poor**	n=2	N/A
	**Mixed**	n=5	N/A
	**Good**	n=19	N/A
**Ascites**	**Yes**	n=30	n=8
	**No**	n=1	n=8

*Age is known for n=12; **CA-125 serum levels have been quantified in n=12;
***At time of analysis, SD = Standard deviation, PFS = Progression free survival, OS = Overall survival
NACT = neoadjuvant chemotherapy, PDS = Primary debulking, N/A = Not applicable

Supplemental Table 2. Individual EOC patient characteristics.

Paper	Age (years)	FIGO stage	PFS (months)*	OS (months)*	Alive	CA125 U/mL (Serum)	CA-125 U/mL (Peritoneal)	NACT/PDS
M01	75	IV	1.5	7.8	No	468	15000	NACT
M02	56	IIIc	6.8	12.5	No	2200	7500	NACT
M03	69	IIIc	20.0	53.8	No	198	1100	PDS
M04	52	IV	4.1	9.4	No	1223	22500	NACT
M05	63	IIIc	20.5	26.2	No	4889	37000	NACT
M06	64	IV	0	8.6	No	1800	4550	NACT
M07	61	IIIc	20.5	55.5	No	2380	26500	PDS
M08	52	IV	5.2	Unknown	Unknown	5700	55000	NACT
M09	59	IIIc	8.4	29.4	No	1304	4450	NACT
M10	45	IV	0	1.7	No	1400	18000	None
M11	69	IIIc	25.1	31.4	No	899	4100	NACT
M12	67	IV	9.1	10.6	No	1560	7000	NACT
M13	76	IIIc	23.9	41.3	No	928	1400	NACT
M14	93	IV	0	1.2	No	412	850	PDS
M15	61	IIIc	Unknown	Unknown	Unknown	10000	12500	NACT
M16	64	IIIc	5.8	37.0	No	352	4650	N/A
M17	52	IV	21.0	31.1	Yes	1621	14000	NACT
M18	63	IIIc	18.0	30.4	Yes	344	1700	PDS
M19	64	IIIc	3.6	29.0	No	31	3250	PDS
M20	47	IIIc	18.9	18.9	Yes	1484	6500	PDS
M21	72	IV	5.7	21.6	Yes	1018	7500	NACT
M22	36	IIIc	22.0	22.0	No	1116	85000	PDS
M23	61	IV	0	14.5	No	10000	95000	NACT
M24	74	IV	1.1	1.1	No	1175	15000	NACT
M25	76	IIIb	22.1	22.1	Yes	1516	18500	NACT
M26	69	IIIc	10.2	18.6	Yes	215	2050	NACT
M27	68	IIIc	14.2	16.5	Yes	1931	2250	NACT
M28	50	IV	0	0.7	No	9909	27000	None
M29	61	IIIc	0	2.5	No	5694	34000	NACT
M30	57	IIIc	2.9	20.3	No	2118	8500	NACT
M31	72	IV	6.9	7.6	No	1500	2900	NACT

*At time of analysis, SD = Standard deviation, PFS = Progression free survival, OS = Overall survival,
NACT = neoadjuva nt chemotherapy, PDS = Primary debulking

Supplemental Table 3. Individual benign patient characteristics.

Number	Age (years)	Fluid type	Pathology diagnosis	Endometriosis	Torsion/infection	CA-125 (U/ml) Serum	CA-125 (U/ml) Peritoneal
B1	53	ascites	Mucinous cystadenoma	-	-	60	750
B2	39	washing	Endometrioma	Yes	-	156	425
B3	62	ascites	Benign fibroma	-	Necrosis	284	500
B4	50	washing	Serous cystadenofibroma	-	-	25	700
B5	41	washing	Serous cystadenoma	-	-	10	80
B6	64	ascites	Serous cystadenoma	-	-	34	550
B7	47	washing	Mucineus cystadenoma	-	-	20	230
B8	65	washing	Mucinous cystadenoma	-	-	23	280
B9	47	washing	Serous cystadenoma	-	-	52	300
B10	41	washing	Mucinous cystadenoma	-	-	15	600
B11	54	washing	Serous cystadenoma	-	-	5	210
B12	69	ascites	Benign not further defined	-	-	2249	1400
B13*	Unknown	ascites	Benign not further defined	Unknown	Unknown	Unknown	435
B14*	Unknown	ascites	Benign not further defined	Unknown	Unknown	Unknown	8000
B15*	Unknown	ascites	Benign not further defined	Unknown	Unknown	Unknown	1050
B16*	Unknown	ascites	Benign not further defined	Unknown	Unknown	Unknown	900

*****Age and CA-125 levels of benign patients B13-B16 are unknown and their benign diagnosis not further defined.

Supplemental Table 4. Antibodies for leukocyte subset panel.

Marker/Dye combination	Manufacturer	Catalog number	Clone	Host species, Isotype	Laser	Filters
CD3 FITC	BioLegend	300406	UCHT1	Mouse IgG1	488	525/40
CD4 BV510	BioLegend	317444	OKT4	Mouse IgG2b	405	525/40
CD8 BUV737	BD Biosciences	612755	SK1	Mouse IgG1	355	740/35
CD45RA PE-Cy7	BioLegend	304126	HI100	Mouse IgG2b	561	763/43
CD197 (CCR7) BV605	BioLegend	353224	G043H7	Mouse IgG2a	405	610/20
CD127 (IL-7Rα) APC	BioLegend	351316	A019D5	Mouse IgG1	638	660/10
CD25 BV786	BD Biosciences	563701	M-A251	Mouse IgG1	405	780/60
CD19 PE-Cy5	Beckman Coulter	A07771	J3-119	Mouse IgG1	561	675/30
CD56 BV711	BioLegend	318336	HCD56	Mouse IgG1	405	710/50
CD16 BUV496	BD Biosciences	612944	3G8	Mouse IgG1	355	525/40
CD45 BUV395	BD Biosciences	563792	HI30	Mouse IgG1	355	385/26
CD33 BV421	BioLegend	303416	WM53	Mouse IgG1	405	450/45
HLA-DR APC-R700	BD Biosciences	565127	G46-6	Mouse IgG2a	638	712/25
CD15 APC-Fire750	BioLegend	323042	W6D3	Mouse IgG1	638	763/40
CD14 PerCP-Cy5.5	Biolegend	301824	M5E2	Mouse IgG2a	488	690/50
CD11b PE	Beckman Coulter	IM2581U	Bear1	Mouse IgG1	561	585/42
CD163 PE-CF594	BD Biosciences	562670	GHI/61	Mouse IgG1	561	610/20
Live/dead ViaKrome808	Beckman Coulter	C36628	N/A	N/A	808	885/40
